# Electrical conductivity as a driver of biological and geological spatial heterogeneity in the Puquios, Salar de Llamara, Atacama Desert, Chile

**DOI:** 10.1038/s41598-021-92105-2

**Published:** 2021-06-17

**Authors:** R. P. Reid, A. M. Oehlert, E. P. Suosaari, C. Demergasso, G. Chong, L. V. Escudero, A. M. Piggot, I. Lascu, A. T. Palma

**Affiliations:** 1 Rosenstiel School of Marine and Atmospheric Science, University of Miami, Miami, FL 33149 USA; 2 Bahamas Marine EcoCentre, Miami, FL 33156 USA; 3 Department of Mineral Sciences, National Museum of Natural History, Smithsonian Institution, Washington DC, 20560 USA; 4 Centro de Biotecnología , Universidad Católica del Norte, Antofagasta, Chile; 5 Departamento de Ciencias Geológicas , Universidad Católica del Norte, Antofagasta, Chile; 6 AP Research Inc., Miami, FL 33157 USA; 7 FisioAqua, Las Condes , 7550024 Santiago, Chile

**Keywords:** Bacteria, Microbial communities, Environmental microbiology, Microbiology, Ecology, Mineralogy

## Abstract

Reputed to be the driest desert in the world, the Atacama Desert in the Central Andes of Northern Chile is an extreme environment with high UV radiation, wide temperature variation, and minimum precipitation. Scarce lagoons associated with salt flats (salars) in this desert are the surface expression of shallow groundwater; these ponds serve as refugia for life and often host microbial communities associated with evaporitic mineral deposition. Results based on multidisciplinary field campaigns and associated laboratory examination of samples collected from the Puquios of the Salar de Llamara in the Atacama Desert during austral summer provide unprecedented detail regarding the spatial heterogeneity of physical, chemical, and biological characteristics of these salar environments. Four main lagoons (‘Puquios’) and more than 400 smaller ponds occur within an area less than 5 km^2^, and are characterized by high variability in electrical conductivity, benthic and planktonic biota, microbiota, lagoon bottom type, and style of mineral deposition. Results suggest that electrical conductivity is a driving force of system heterogeneity. Such spatial heterogeneity within the Puquios is likely to be expanded with temporal observations incorporating expected seasonal changes in electrical conductivity. The complexity of these Andean ecosystems may be key to their ability to persist in extreme environments at the edge of habitability.

## Introduction

Life on Earth can thrive in almost every ecological niche, and many extremophiles prosper in the harshest of environments^1–6^. Study of modern extreme environments suggests that variability in physico-chemical conditions can induce differences in microbial morphology, ecosystem function, and mineral deposition^7–9^. Detailed characterization of the response of ecosystem function and microbe-mineral interactions to variations in the physico-chemical conditions that are commonly observed in extreme environments are necessary for accurate interpretations of depositional setting and potential biosignatures in the rock record^10,11^.

Extreme environments hosting microbial life are common in the Andean regions of South America. Studies of more than 40 microbial ecosystems forming in harsh conditions in Chile, Argentina, and Bolivia document a wide range of geological, chemical, biological and mineralogical processes^12–33^. An example of a gypsum-depositing system in an extreme Andean environment is a series of lagoons locally known as Puquios in the Salar de Llamara, located in the Atacama Desert. The Salar de Llamara is located in one of the main endorheic basins in the Pampa del Tamarugal, Central Depression, of the Norte Grande de Chile^34–36^. The Puquios are variably sized depressions filled with ground water. The Pampa del Tamarugal is the largest and last base level before the Pacific Ocean for the sedimentary fill and water input provided by the High and Preandean Cordilleras. The sedimentary fill consists of detrital sediments and volcanic rocks that can reach up to 1300 m thickness^37^. The Puquios, in this arid to hyperarid environment of the Atacama Desert, are subject to a complex interplay of physical, chemical, geological, and biological processes. Known environmental pressures in the Salar de Llamara include high UV irradiation, extreme aridity, and significant fluctuations of temperature and salinity^38,39^, all of which contribute to the characterization of the Puquios as a dynamic extreme environment^40^_._ Despite these harsh environmental conditions, the Puquios support the development of a variety of biotic communities and exhibit diverse styles of gypsum deposition. Previous studies of these ecosystems have typically focused either on the brine chemistry, or the microbial communities, or the deposition of carbonate and evaporitic minerals^12,13,19,22,41–43^, with limited holistic characterization across disciplines. In order to more fully understand these extreme settings, a multidisciplinary approach that captures physical, chemical, geological, and biological observations is needed.

This paper presents data from multidisciplinary studies of the Puquios in the Salar de Llamara during the austral summer. Results provide unprecedented detail regarding the spatial heterogeneity of physical, chemical, and biological characteristics of these salar environments. Our observations include more than 1285 in situ measurements of electrical conductivity in the surface and bottom waters and descriptions of free-living biota in each lagoon, endolithic microbial communities, and mineralogic and morphologic analyses of bottom types. Spatial distributions of biological and geological features observed throughout the system appear to be linked to gradients in electrical conductivity. The high degree of spatial heterogeneity in all measured parameters highlights the need for multi-disciplinary studies across space and time to understand ecosystem function and mineral deposition in extreme environments.

### Environmental setting

**Figure 1:**
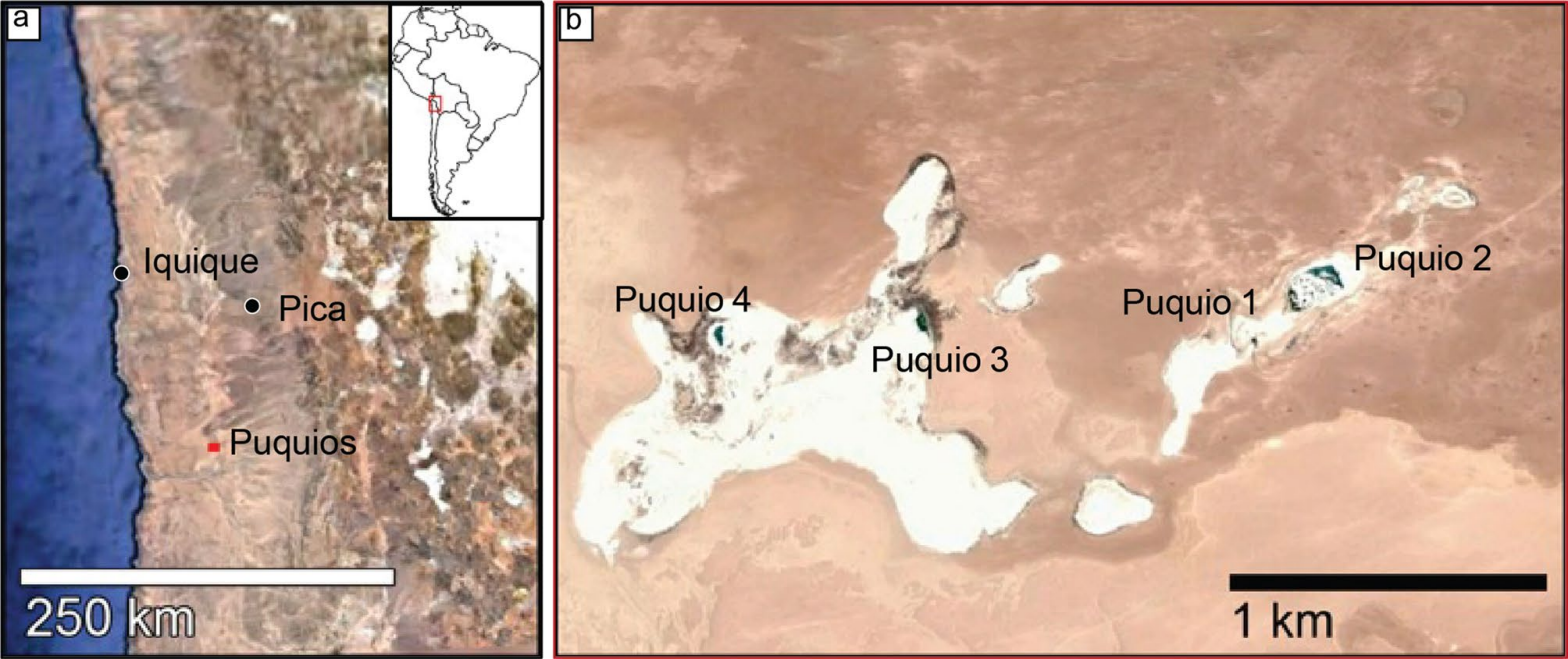
Location map—(**a**) regional context and location of the Puquios within northern Chile; (**b**) enlarged view of the red square in (**a**) showing the location of the Puquios within the Salar de Llamara. Imagery from ArcGIS World Imagery basemap; sources: Sources: Esri, DigitalGlobe, GeoEye, i-cubed, USDA FSA, USGS, AEX, Getmapping, Aerogrid, IGN, IGP, swisstopo, and the GIS User Community. Figure was created in Microsoft PowerPoint for Mac v16.48 https://www.microsoft.com/en-us/microsoft-365/powerpoint.

The Puquios (Fig. 1) are located in the eastern part of the Salar de Llamara (21° 23′ S–69° 37′ W), an actively depositing Salar (salt flat) in the Pampa del Tamarugal, Central Depression^13^. This salar is located in the distal part of the Arcas Alluvial Fan that provides most of the basin input^44^. The hyperaridity of the Central Depression has fostered the deposition of evaporitic sediments since at least the Middle Miocene^36^, and is controlled by the geologic and oceanographic setting^36,45–47^. Rainfall is extremely low, estimated to be less than 1 mm in the Atacama Desert core^48^ as a result of the rain-shadows cast by both the Coastal Cordillera and the High Cordillera. Furthermore, evaporation rates significantly exceed rainfall, with up to 90% of rainfall in the Western Cordillera lost via evaporation and runoff^49^. The permanent surfacing of sub-terrain waters is recharged by a combination of summer monsoons (Invierno Altiplánico or Invierno Boliviano) in the High Cordillera^50–52^ that continuously drain through the alluvial fan systems punctuating the eastern margin of the basin^36,53,54^ with occurrences of coastal dripping fog^55,56^. The combination of these hydrological processes associated with the alluvial fan systems and the geomorphological characteristics at the boundary between the Coastal Cordillera and the Pampa del Tamarugal allows the shallow ground water levels to create the small lagoons called puquios with their biological communities. Brines in the Puquios are managed by SQM Ltd.; environmental protection measures were enacted in 2012 to preserve brine chemistry and water levels in the salar environment^22,42,57,58^.

**Figure 2:**
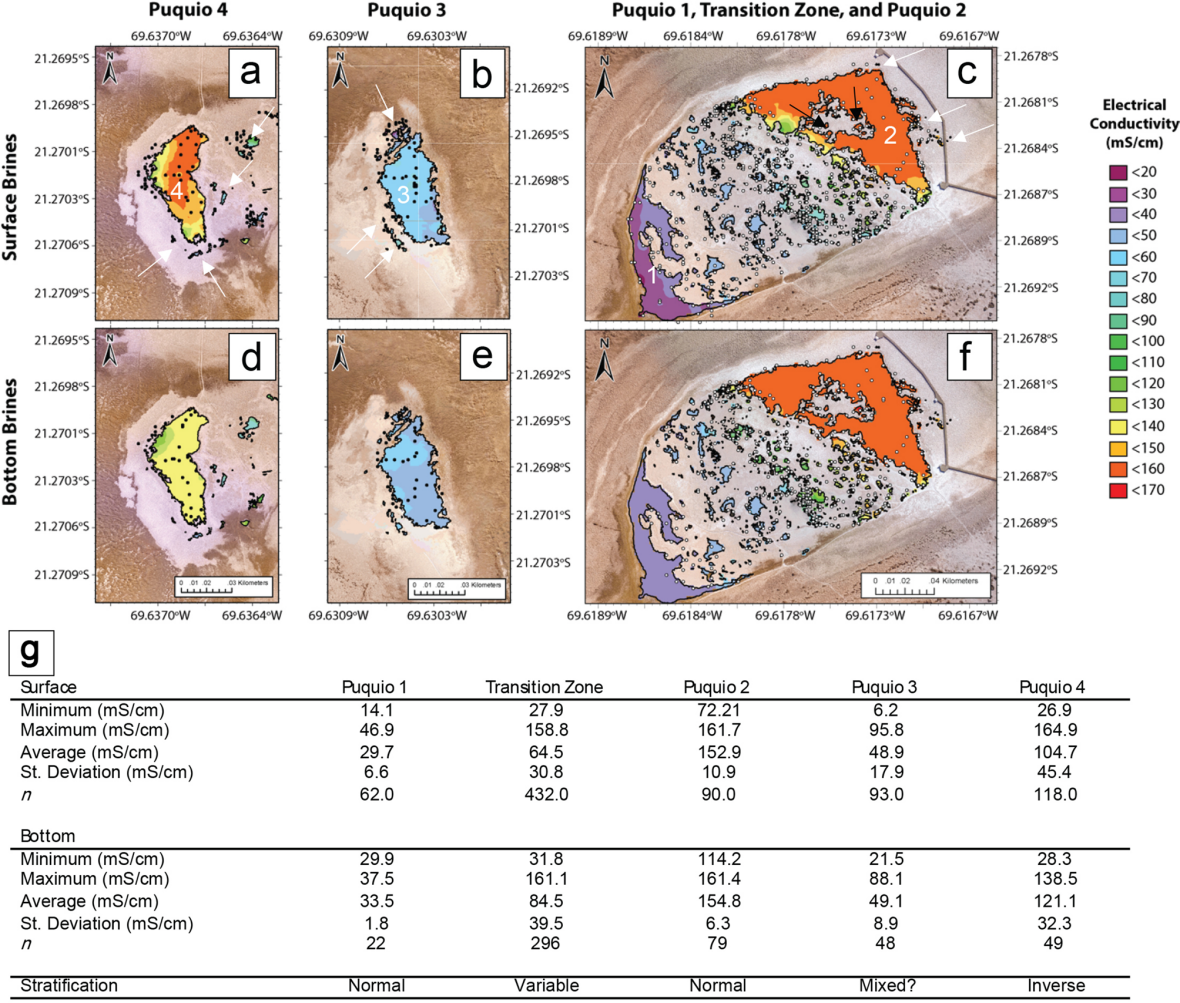
Contour maps of in situ electrical conductivity (mS/cm) in the Puquios. Measurement locations are shown as black dots (Puquios 3 and 4), and white dots (Puquios 1, 2, and the Transition Zone). Scale of all maps is 1:1180 km. Surface brine measurements are shown in (**a–c**) and bottom brine measurements are shown in (**d–f**). Maps were created in ArcGIS Pro https://www.esri.com/en-us/arcgis/products/arcgis-pro. Minimum and maximum measurements of electrical conductivity (mS/cm) in the surface and bottom brines of each of the Puquios during the November 2017 field campaign are shown in (**g**). Stratification was assessed following the methods of Babel (2004) and refers to the main lagoon waters and the smaller peripheral lagoons.

The Puquios system is characterized by four main lagoons (Figs. 1b, 2), plus a series of smaller peripheral ponds (ranging from sub-meter to tens of meters in diameter). Puquio 1 consists of a main shallow lagoon with low-turbidity waters, an area of 1760 m^2^ and a maximum depth of 50 cm. A multitude of small ponds exist between the main lagoons of Puquio 1 and 2 that are collectively referred to as “the Transition Zone”. The Transition Zone contains nearly 400 small lagoons of various water turbidities, sizes, and depths (maximum depth recorded 80 cm). Puquio 2 is largest lagoon in the Salar de Llamara with very low-turbidity brines, an area of 4650 m^2^ and a depth less than 1 m. The main lagoon of the Puquio 3 system has an area of 1660 m^2^_,_ elongated north–south, surrounded by a number of smaller, peripheral ponds. Water in Puquio 3 is clear, whereas the shallow peripheral ponds often have milky-white to peach-colored water. Puquio 4 consists of one main lagoon (1485 m^2^) elongated north–south, surrounded by numerous peripheral ponds, mostly to the east. The main lagoon of Puquio 4 appears turquoise in color, and the water is clear, whereas the surrounding ponds also often have milky-white to peach-colored water similar to those adjacent to Puquio 3.

## Results

Results from high resolution studies of electrical conductivity of brine waters, free living biota, microbial endoliths, and bottom types are presented below.

### Electrical conductivity

More than 1285 in situ measurements of electrical conductivity (EC, Fig. 2, Supplemental Table S1) were collected from both the surface brines and depths of the lagoons in the Puquios to characterize environmental gradients; EC is interpreted to be a proxy for the salinity of the lagoon water. In situ measurements reveal chemical gradients on multiple scales, documenting spatial heterogeneity not only within the Puquios system as a whole, but also defining a high degree of variability within a single lagoon.

Within the system as a whole, EC exhibited a high degree of spatial heterogeneity between Puquios (Fig. 2, Supplemental Fig. S1, Supplemental Table S1). The lowest EC value was observed in the surface brine of a peripheral lagoon of Puquio 3 (6.2 mS/cm, Fig. 2b,g), and the highest EC values were observed in the surface brine of the main lagoon of Puquio 4 (164.9 mS/cm, Fig. 2a,g). Spatial trends in surface EC were also observed within the Puquios 1 and 2 system, where EC values grade from lower values in the main lagoon of Puquio 1 to significantly higher EC values in the main lagoon of Puquio 2 in the surface brine (Fig. 2c,g). Observation of such variability in the Puquios system suggests that hydrological connections between each of the four Puquios may not be direct, and that higher EC values reflect higher degrees of evaporation and/or lower rates of groundwater flux into the brine pools.

Spatial heterogeneity of surface EC values was also commonly observed within individual lagoons. The main lagoons of Puquios 2, 3, and 4 contain ranges of EC values greater than 89.5, 89.6, and 138.0 mS/cm in their surface brines (Fig. 2a–c,g). EC values of surface brines in the Transition Zone between Puquios 1 and 2 exhibit a range of more than 130.9 mS/cm (Fig. 2c,g). Comparison of surface brine EC measurements in the peripheral lagoons also demonstrated a large range in measured values across short distances, suggesting that the peripheral ponds can be isolated from the main lagoon water, and as a result may develop distinct EC values and degrees of spatial heterogeneity.

Gradients in EC measurements were also a common characteristic of bottom brines in the Puquios. Like the surface brine measurements, the highest bottom brine EC value (161.4 mS/cm) was observed in Puquio 2 (Fig. 2f,g), while the lowest EC measurement (21.5 mS/cm) was recorded in the bottom waters of Puquio 1 (Fig. 2f,g). Spatial heterogeneity of EC values in the bottom brines was reduced relative to the range observed in surface brines (Fig. 2c,f,g).

Differences between the measured EC values in the surface and bottom brines indicate that the pools were not only spatially heterogeneous, but also vertically stratified. Vertical stratification was defined following Babel^59^, where normal stratification is indicated by bottom values exceeding surface values, and inverse stratification is indicated by bottom values being lower than surface values. Results are presented in Fig. 2g. Notably, stratification also exhibited spatial heterogeneity: Puquios 1 and 2, much of the Transition Zone, as well as peripheral ponds in the vicinity of Puquios 2 and 3 exhibited normal stratification, whereas measurements in the main lagoons and peripheral ponds near Puquio 4 were inversely stratified, and Puquio 3 contained a well-mixed water column at the time of measurement (Fig. 2g, Supplemental Fig. S1).

### Free-living biota

The free-living biota that thrives in the aquatic component of the Puquios system is comprised of four major groups: phytobenthos (microalgae that exist on the bottom), phytoplankton (microalgae in the water column), zoobenthos (invertebrates found on the bottom or buried within the first few centimeters of substrate) and zooplankton (invertebrates in the water column). The first two groups, which are photosynthetic primary producers, are represented mainly by diatoms (50 species) and by the less diverse cyanobacteria (8 species). The primary and secondary consumers present on the bottom and in the water column are represented by 8 taxa: arachnids (spiders), annelids (worms), branchiopods (brine shrimps), coleopterans (beetles), copepods, dipterans (flies), hexapods (springtails), gastropods (snails) and odonates (dragonflies). Several of these taxa have complex life cycles and thus occur in different ontogenetic stages (i.e., from larval stages to adults). Some, like dipterans and odonates, only spend their larval phase in the water while some, like the brine shrimp *Artemia franciscana*, are holoplanktonic.

**Figure 3:**
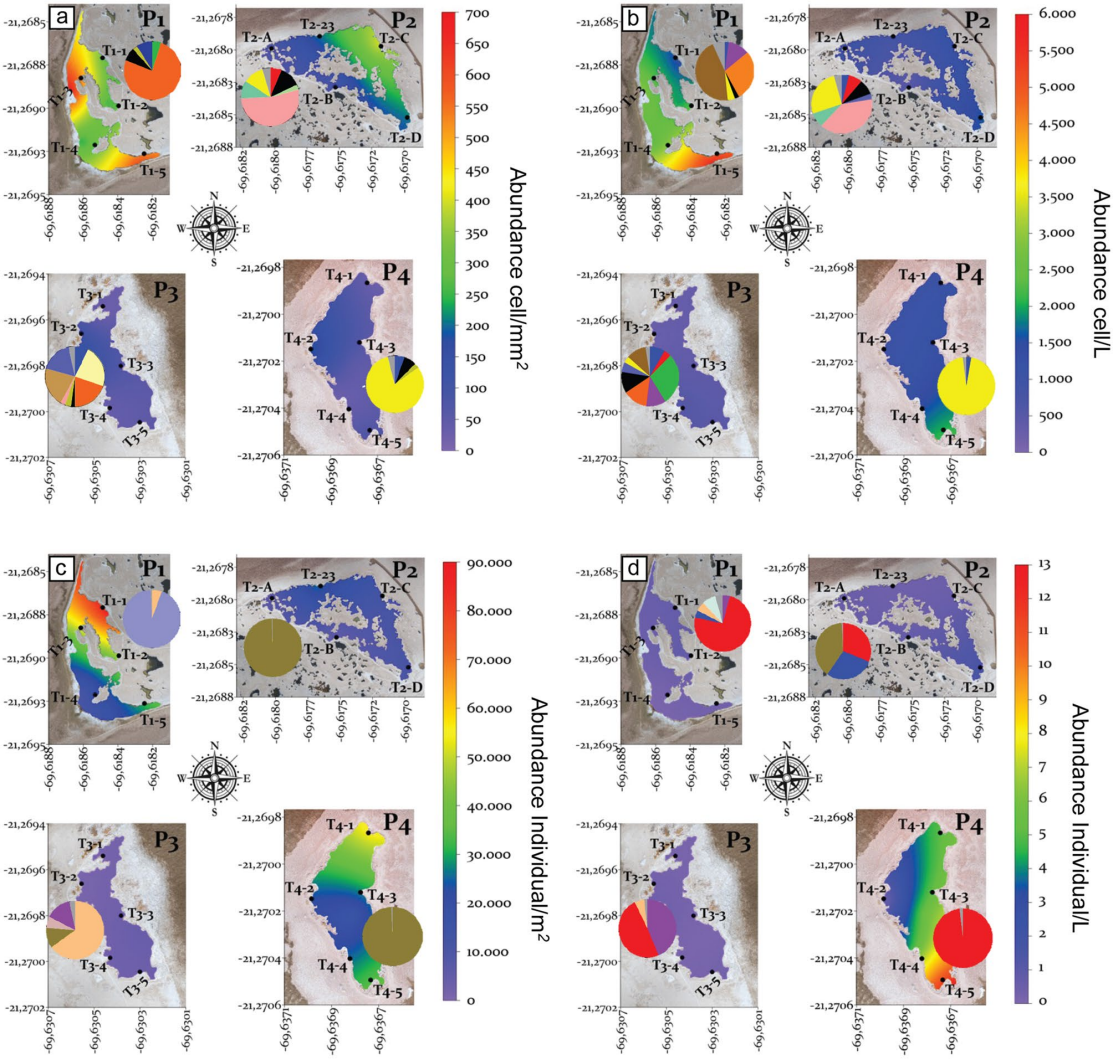
Abundances of the main biotic assemblages present in the bottom and water column of each puquio, with ranges defined by color bar legend to the right of each group: (**a**) phytobenthos, (**b**) phytoplankton, (**c**) zoobenthos and (**d**) zooplankton. Pie charts show the diversity and relative abundance of corresponding taxa within each lagoon. Main taxa are listed in decreasing order of abundance: (**a**) Phytobenthos; P1: *Denticula, Achnanthidium*, Fragilariaceae, P2: *Staurosira, Staurosirella*, Fragilariaceae, *Nitzschia,* P3: *Denticula, Brachysira, Mastogloia*, Cyanophyta, P4: *Nitzschia, Achnanthidium*, Fragilariaceae, (**b**) Phytoplankton; P1: Cyanophyta, *Denticula*, *Campylodiscus*, P2: *Staurosira, Nitzschia, Staurosirella*, Fragilariaceae, *Amphora*, P3: *Brachysira, Denticula*, Fragilariaceae, *Campylodiscus*, P4: Nitzschia, (**c**) Zoobenthos; P1: Gastropoda (*Heleobia*), Ceratopogonidae, P2: Ephydridae, P3: Ceratopogonidae, Arachnida, Ephydridae, P4: Ephydridae and (**d**) Zooplankton; P1: Branchiopoda (*Artemia*), Hexapoda, Ceratopogonidae, P2: Ephydridae, Branchiopoda (*Artemia*), Copepoda, P3: Branchiopoda (*Artemia*), Arachnida, Ceratopogonidae, P4: Branchiopoda (*Artemia*). Maps were generated using Surfer v13 https://www.goldensoftware.com/products/surfer.

All four assemblages of biota exhibited ample variability in several ecological descriptors (e.g., number of taxa and Shannon diversity index, Supplemental Table S2), both within and between Puquios. The abundance of many species within each assemblage exhibited notable differences between Puquios and the predominance of one or few taxa was common (see pie charts in Fig. 3). For example, *Denticula*, *Staurosira* and *Nitzschia* are the most represented genera of diatoms on the bottom of Puquios 1, 2 and 4, respectively, while the water column is dominated by Cyanophyta in Puquio 1 and *Staurosira and Nitzschia* in Puquios 2 and 4, respectively. Although, the taxa recorded in the four Puquios are commonly observed in other high Andean saline systems^60^, the particular dominance of certain taxa in different puquios is intriguing. For example, the genus *Nitzschia* contains many pollution-tolerant species^61^ that have been used as indicators of water quality^62^.

Between two to three taxa represented > 75% of the richness for phytobenthos and phytoplankton in P1, P2, and P4 and for zoobenthos and zooplankton in all four puquios. The abundance of phytobenthos ranged from a few cells/mm^2^ to ca. 700 cells/mm^2^ and was highest in Puquio 1, which also exhibited important differences among sampling points, with the next highest abundance of phytobenthos observed in Puquio 2. Phytobenthos was least abundant in Puquios 3 and 4 (Fig. 3a). Phytoplankton exhibited an even more pronounced pattern of abundance variation between and within Puquios. The highest abundance of phytoplankton was recorded in the southern-most sampling point of Puquio 1, more than twice that found on its northern-most point. Puquios 2, 3, and 4 shared lower abundances in the range of 1000 cells/L (Fig. 3b). The abundance of zoobenthos was also greater, and highly variable in Puquio 1, with up to 90,000 individuals/m^2^ in its northern section and around 20,000 individuals/m^2^ along its southern edge. Puquio 2, followed by 3, displayed the lowest abundance, and Puquio 4 exhibited a varied and intermediate level of abundance (Fig. 3c). Zooplankton displayed a consistently low abundance in Puquios 1, 2, and 3, in contrast to Puquio 4, which was characterized by a relatively higher abundance of zooplankton, particularly in its southern-most section (Fig. 3d).

A linear regression analysis of electrical conductivity and the abundance of each biotic assemblage, as well as the Shannon diversity index (H′), was conducted (Supplemental Fig. S2). There was a significant negative relationship between EC and Shannon diversity (H′) among the phytoplankton, phytobenthos, and zoobenthos (Supplemental Fig. S2). There was no significant relationship between zooplankton diversity and EC (Supplemental Fig. S2). There was a negative relationship with phytobenthos abundance and increasing electrical conductivity (Supplemental Fig. S2). There was a positive relationship with zooplankton abundance and electrical conductivity. No relationships between EC and the cellular abundances of phytoplankton and zoobenthos were observed.

### Bacterial community analysis

**Figure 4:**
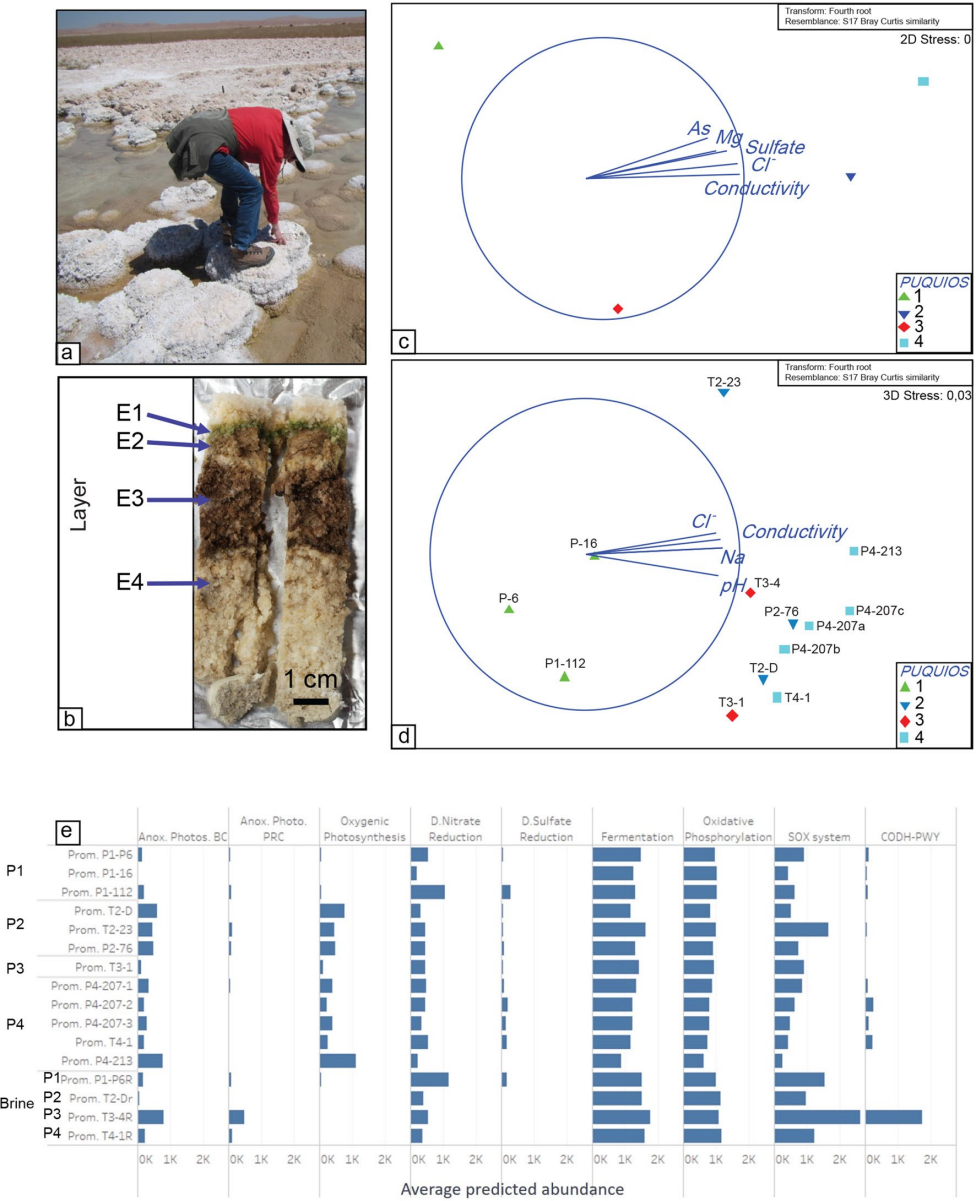
Microbial community variability in subaerial structures and associated brines. Photographs showing the area sampled from subaerial structures surrounding the Puquios (**a**) and the E1 layer sampled within the mini-core for analysis (**b**). Non-metric multidimensional scaling for OTUs with vectors overlay showing correlation of environmental variables in the brines (**c**) and E1 layer of subaerial structures (**d**). Relative abundance of microbial metabolisms as predicted by PICRUst is shown in (**e**).

The bacterial communities were assessed in subaerial gypsum structures and the adjacent brines (Fig. 4a). The endolithic bacterial communities inhabiting the subaerial gypsum-halite structures surrounding the Puquios exhibited a layered pattern at each sampling location, with a gradient of colors changing from green (layer E1) to brown (layers E2 and E3) to colorless (layer E4) with increasing depth observed in hand sample (Fig. 4b). Samples of the E1 bacterial communities from twelve gypsum structures as well as the free-living microbial communities collected in four brine samples were analyzed by the 16S rRNA gene bacterial amplicon sequencing. The average diversity indices of the bacterial communities in the brines were 3.9 (P1), 3.1 (P2), 3.1 (P3), and 2.6 (P4). The average diversity indices in the E1 layer were 4.6 (P1), 4.73 (P2), 4.75 (P3), and 3.5 (P4). The evenness index was less than 0.82 for the E1 layer and less than 0.61 for brine samples. The similarity between the E1 samples was > 40% at the phylum level and > 25% at the genus level. Predominant bacteria in the E1 layer and brines affiliate mostly to high-rank prokaryotic taxa, Proteobacteria, Planctomycetes, Verrucomicrobia, Cyanobacteria, and Actinobacteria. Within the Proteobacteria phylum, the alpha (orders Rhodobacterales, Rhizobiales) and gamma (orders Alteromonadales, Chromatiales) classes were predominant. The similarity among the brine samples was > 55% at the phylum and > 30% and the genus level. Bacteria in these groups affiliate mostly to the high-rank prokaryotic taxa Proteobacteria and Bacteroidetes, as previously reported^39^. Classes alpha (order Rhodobacterales and Sphingomonadales) and gamma (orders Chromatiales and Oceanospirillales) were predominant within the Proteobacteria phylum.

A non-Metric Multi-dimensional scaling (nMDS) analysis grouped the brine bacterial communities related to brine chemistry (EC, Cl^−^, and SO_4_^2−^, Pearson correlation > 0.9; Supplemental Table S3), grouping Puquio 1 away from the other higher salinity locations (Fig. 4c). Differences in the brine microbial communities were attributed to the genera of anoxygenic phototrophic bacteria from Alpha and Gammaproteobacteria and Chloroflexi groups (Pearson > 0.9, Supplemental Fig. S3). E1 layer bacterial communities sampled from gypsum structures were also grouped related to lagoon water chemistry, displaying a strong correlation with EC, pH, and Na^+^ concentrations (Pearson correlation > 0.8) (Supplemental Table S3, Fig. 4d). E1 samples from Puquio 1 contained higher relative abundances (Pearson correlation > 0.85) of sulfate reducing bacteria (e.g. Desulfobacterales) and Chloroflexi (Supplemental Fig. S4a,b). Puquios 2, 3, and 4 were characterized by higher EC brine values and higher abundances of sulfur oxidizing Alphaproteobacteria (Rhodospirillaceae and Hyphomicrobiaceae) and of the halophilic and halotolerant genera of Cyanobacteria (Chroococcales and Oscillatoriales) within the E1 layers (Supplemental Fig. S3d).

The metabolisms from the predicted metagenomes by PICRUSt (Phylogenetic Investigation of Communities by Reconstruction of Unobserved States) suggests that there are key metabolic differences between E1 layers and the free-living communities in the brines, and also between the E1 layers from each Puquio. The occurrence of the sulfur oxidation pathway appears to be dominant in both brines and subaerial structures (Fig. 4e). Phototrophy is more abundant in E1 samples, whereas heterotrophic and aerobic metabolisms are more important in the brine samples (Fig. 4e). There is also a clear differentiation between the subaerial structures. Anoxygenic and oxygenic photosynthesis are more abundant in E1 from the most saline ponds. Meanwhile, nitrate and sulfate reduction and the use of CO and H_2_ as electron donors are more represented in E1 from the lowest salinity pond (Puquio 1). Desulfobacterales was observed among the sulfate reducers which has been reported previously in the subaqueous structures^14^. Desulfobacterales was registered as a dominant, active sulfate reducing bacteria and primary hydrogenotrophs in the upper layer of a microbial mat^63^.

### Bottom types and mineral precipitation

**Figure 5:**
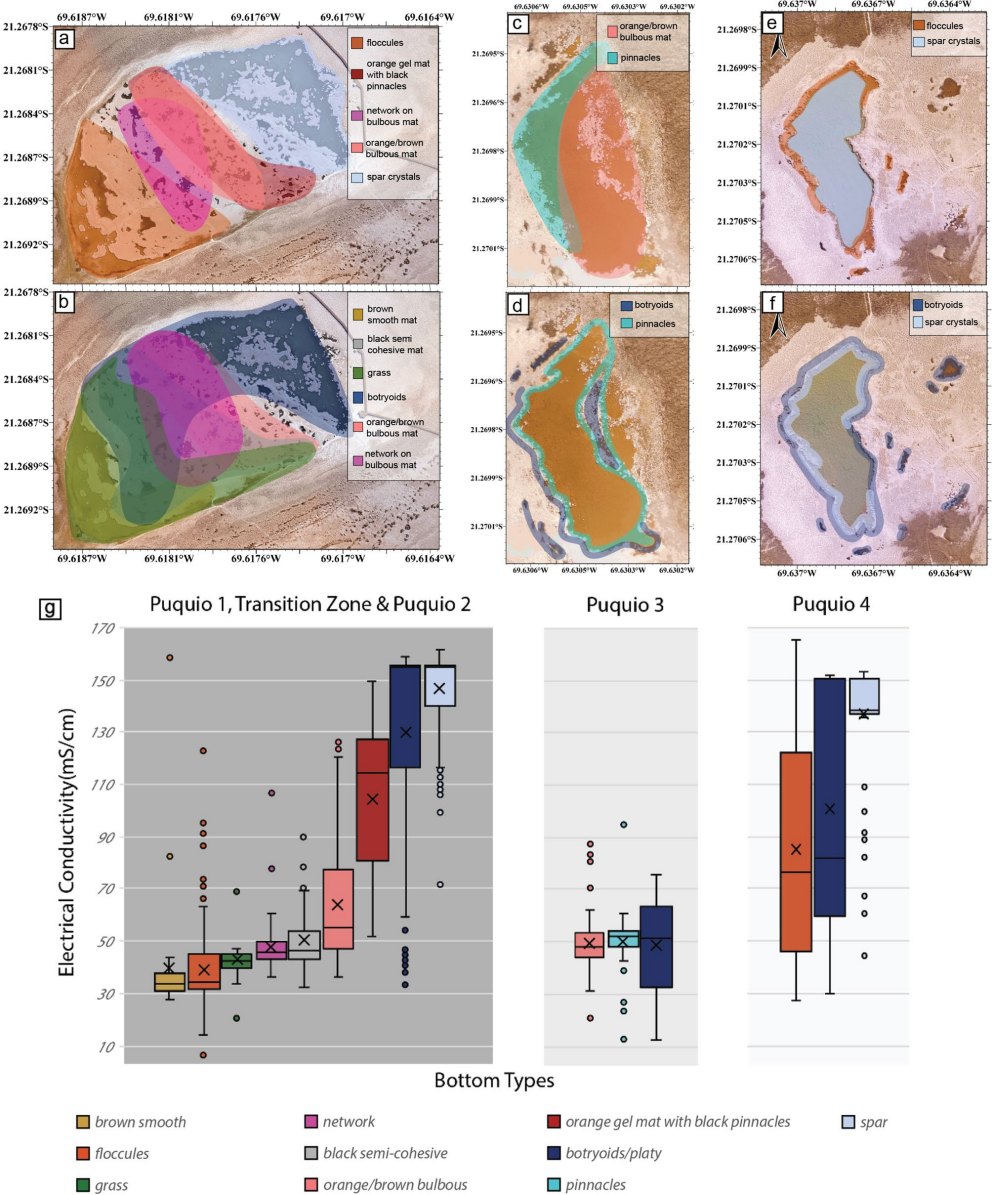
Generalized maps showing geographic trends of principle bottom types (PBT), generated from individual point maps (Supplemental Fig. S6). General location of dominant (**a**) bottom surfaces and (**b**) side wall surfaces for Puquios 1 and 2; general location of dominant, (**c**) bottom surfaces and (**d**) side wall surfaces for Puquio 3; general location of dominant, (**e**) bottom surfaces and (**f**) side wall surfaces for Puquio 4. Maps were generated in Global Mapper v20.1 https://www.bluemarblegeo.com/. Zones were traced using data from Supplemental Fig. S6 using Adobe Illustrator 2021 https://www.adobe.com/products/illustrator.html. (**g**) Box and whisker plots showing the correlation of bottom and side types with electrical conductivity in mS/cm.

**Figure 6:**
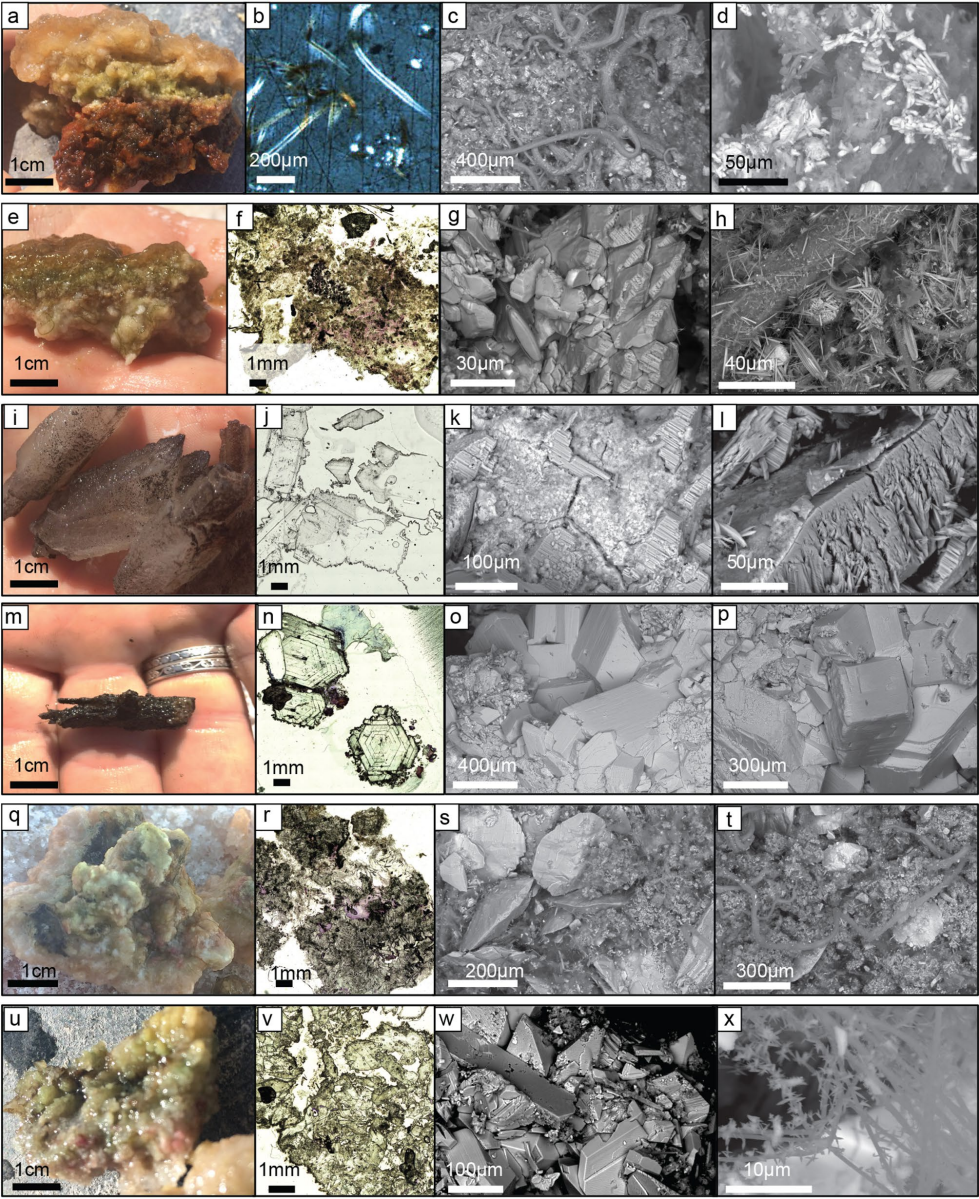
Microbe-mineral interactions—First column is the field photo of the hand sample. Second column is a photomicrograph of petrographic “wet” thin section, stained with crystal violet to highlight gram positive bacteria. Third and fourth column are SEM photos of critically dried samples. Orange/brown bulbous mat PBT from transition zone between Puquio 1 and 2 (**a**–**d**). (**b**) Photomicrograph of “wet” thin section under cross-polarized light showing Mg-silicates forming along cyanobacterial sheaths; (**c,d**) images showing microcrystalline gypsum crystals in an EPS matrix with cyanobacterial filaments. Orange gel mat PBT from transition zone between Puquio 1 and 2 (**e–h**). (**e**) Hand sample showing orange gel, green and purple microbial layers, and granular minerals; (**g,h**) images showing gypsum crystals in an EPS matrix (**g**) and acicular crystals coating a cyanobacterial filament (**h**). Spar crystals PBT sampled from a small lagoon near Puquio 2 (**i–l**). (**j**) coarse gypsum crystals, often with jagged outer surfaces with dark coatings shown in petrographic thin section; (**k,l**) images show large flat crystal faces coated with a thin crust, rich in manganese (EDS). Spar crystals PBT sampled from a small lagoon near Puquio 2 (**m–p**). (**n**) Cross sections of euhedral prismatic gypsum crystals with dark horizons; (**o,p**) images show large blocky, flat crystal faces, coated with granular aggregates rich in manganese (EDS). Pinnacles PBT sampled along the northern margin of Puquio 3 (**q–t**). (**r**) Gypsum is microcrystalline with an adjacent strip of lenticular sparry gypsum and blocky to prismatic gypsum; (**s**) images showing lenticular gypsum embedded in EPS and fine-grained gypsum (**t**) and filamentous cyanobacteria with gypsum crystals on sheaths. Botryoids PBT sampled from within Puquio 4 (**u–x**). (**v**) Crystalline matrix of prismatic gypsum; edges are coated with a fine microcrystalline crust; (**w**) images showing blocky crystals with planar faces and (**x**) fine cyanobacterial filaments with stellate crystals adhering to the sheaths.

A range of different bottom types (Fig. 5, Supplemental Figs. S5, S6) and corresponding variations in mineral precipitation (Fig. 6) were observed within the Puquios. The bottom of Puquio 1 was dominated by loose flocculent sediment (Supplemental Figs. S5k, S6a, S7a), lacking lithified mineral structures with topographic relief. Scanning Electron Microscopy/Energy Dispersive X-Ray Spectroscopy (SEM/EDS) analyses documented abundant small granular crystals of gypsum within the flocculent material, often forming aggregations. Carbonates and magnesium clays were also present. Microbes and exopolymeric substance (EPS) were intimately associated with the minerals in Puquio 1. Diatoms and gastropods were found in abundance.

Bottom types in the Transition Zone were highly variable, grading from flocculent sediment to a variety of orange, brown, and black microbial mats, with various morphologies (Fig. 5, Supplemental Figs. S5, S6a–c). SEM analyses of critically dried samples of mat types throughout the Transition Zone (Fig. 6a–h) showed intimate mixtures of EPS, microbes and diatoms with fine-grained minerals (primarily gypsum), often with distinct lamination of the mineral substrate. Gypsum crystals within the microbial mats exhibited various habits including lenticular, anhedral, irregular etc., and small crystals often aggregated into larger crystals within organic matrices (Fig. 6c,d,g). Accumulations of minerals and microbes were present in most samples examined. Another striking characteristic of samples collected from ponds in the transitional zone was the common appearance of small acicular crystals accruing along filamentous cyanobacterial sheaths (Fig. 6h). Additional mineral accumulations found in abundance throughout the Transition Zone included various carbonate species, sodium sulfates, magnesium clays, and manganese oxides.

Euhedral gypsum spar characterizes the bottom type of the main lagoon of Puquio 2 as well as the Transition Zone lagoons to the southwest (Figs. 5a,b, 6i–p, Supplemental Figs. S5aa,ab, S6a,b, S7d). Samples displayed cm-scale selenite crystals and lacked organic matrices. These large crystals had well-developed crystal faces, lacked agglutinated grains, and were sometimes coated with manganese oxides (Fig. 6m). The side walls of Puquio 2 were comprised mainly of gypsum botryoids, which often merged into plates. Diatoms were common.

The main lagoon of Puquio 3 was dominated by pinnacles (Fig. 5c,f, Supplemental Figs. S5v,w, S6c,d, S7e) along the northwestern margin of Puquio 3, and orange/brown microbial mats in the southeast (Fig. 5c, Supplemental Fig. S5t). SEM/EDS analyses revealed granular and laminated gypsum accumulations closely associated with microbes (Supplemental Table S4, Fig. 6q–t). A sample collected in the south exhibited larger crystals and aggregates, whereas a sample collected in the north of Puquio 3 (Fig. 6r) was largely microcrystalline.

The dominant bottom type in Puquio 4 was gypsum spar (Figs. 5e,d, 6q–t, Supplemental Figs. S5y, S6e,f). Where water circulation was poor around the margins, floccules were common (Fig. 5f, Supplemental Fig. S6e). A sample collected from the northeast margin of Puquio 4 (Fig. 6u–x, Supplemental Table S4) contained well-cemented aggregates of coarse (> 100 µm) crystals of gypsum. Although not abundant in the sample, microbes and EPS were also present in minor amounts.

## Discussion

**Figure 7:**
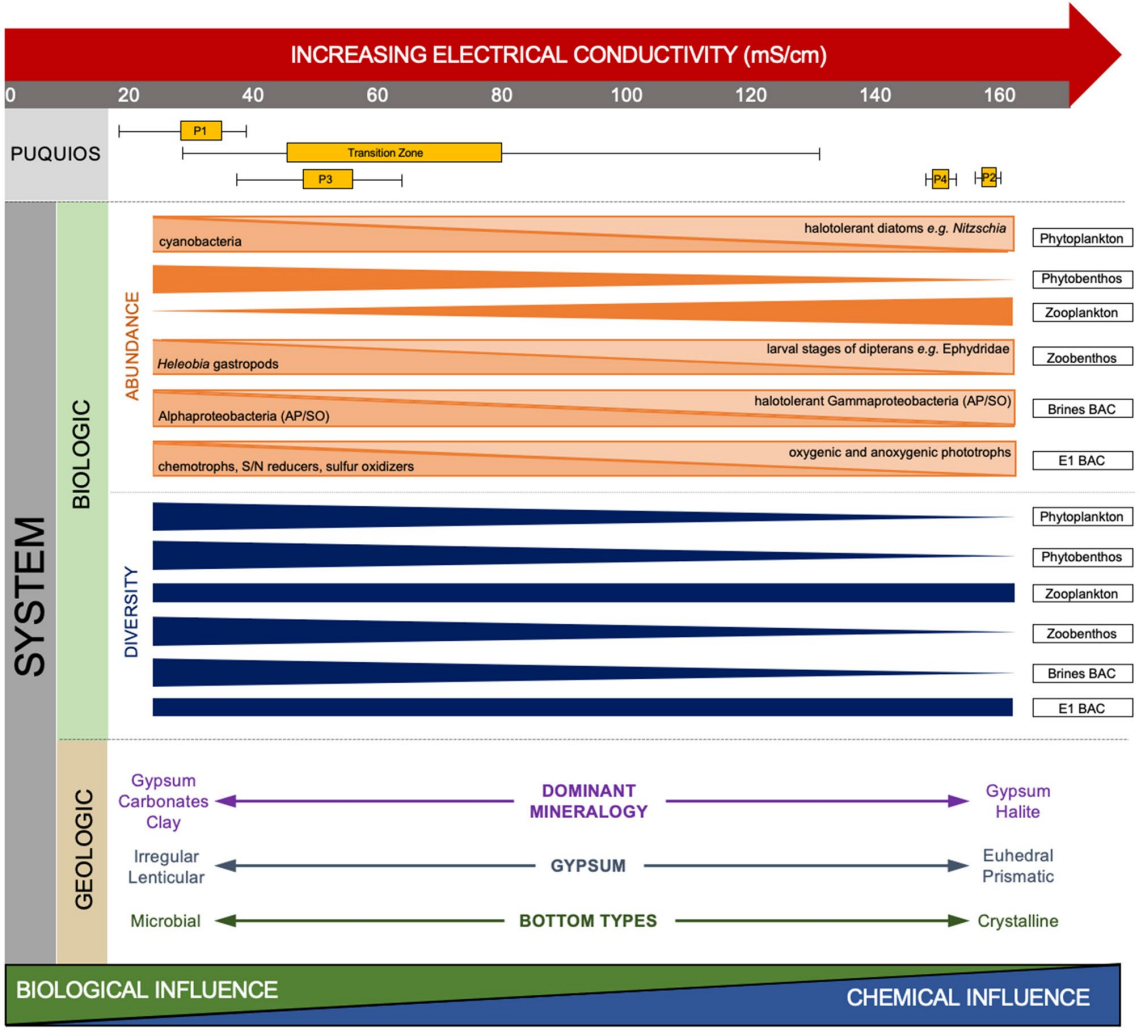
Summary diagram showing a conceptual framework of the trends observed in the biologic and geologic components of the Puquio system across an increasing gradient in electrical conductivity. Brine BAC refers to bacterial communities in the brines. E1 BAC refers to bacterial communities in the E1 layer. Thickness of diversity and abundance bars indicate general trends as supported in Figs. 3, 4 and Supplementary Figs. S2–S4. In cases where total abundance showed no apparent relation with EC (e.g. Phytoplankton, Zoobenthos, Brines BAC, and E1 BAC), the bars were subdivided to highlight the effect of EC on individual functional groups. *AP* anoxygenic phototrophs, *SO* sulfur oxidizers, *S* sulfate, *N* nitrate. Figure was created in Microsoft PowerPoint for Mac v16.48. https://www.microsoft.com/en-us/microsoft-365/powerpoint.

Heterogeneity within and between Puquios is reflected across all parameters investigated, including electrical conductivity, diversity and abundances of free-living biota, community structure and metabolisms of endolithic microbial ecosystems, as well as the dominant lagoon bottom types and associated minerals. For the most part, variations in these ecological, microbiological, and mineralogical datasets appear to follow gradients of electrical conductivity, which exhibits heterogeneity on both the scale of the salar environment as a whole, as well as within single lagoons. As summarized in Fig. 7 and discussed below, diversity and abundance of phytobenthos, zoobenthos, phytoplankton, zooplankton, and microbial communities vary across a gradient of increasing EC. Within the EC gradient, a dynamic interplay of microbial, ecological, and mineralogical processes is hypothesized to provide ecosystem flexibility, allowing biota in the Puquio system to adapt to the high degree of variability of environmental conditions that characterize these extreme environments.

Gradients in electrical conductivity are the predominant unifying factor that drives the ubiquitous spatial variability throughout the lagoons (Fig. 7). New insight into the degree of spatial heterogeneity of EC values across the Puquios system was gained by including measurements at two depths from multiple locations around the margins of each of the main lagoons, as well as in each of the surrounding peripheral ponds. A large gradient in EC values (6.2–164.9 mS/cm) and variability in the degree of stratification in the brines was observed within the system as a whole, as well as within individual lagoons (Fig. 2, Supplemental Fig. S1). Observations of heterogeneity within a single lagoon indicate that the mechanisms generating such spatial variability occur on time scales shorter than processes like wind-driven physical mixing can homogenize the lagoon waters. Further high-resolution studies are required in order to identify specific mechanisms driving spatial heterogeneity within a single lagoon. Previous studies have found similar large-scale gradients in EC values in the Puquios system as a whole, primarily in the main lagoons and peripheral ponds in the vicinity of Puquio 1, the Transition Zone, and Puquio 2^13,28,30,41–43^. EC measurements in these previous studies, as well as in situ measurements of total dissolved solids (TDS) conducted by Otálora et al.^22^ documented a trend of increasing EC moving from SW to NE in the P1-TZ-P2 region, which is consistent with hydrogeological models of flow direction in the area^57,58^. Variations in the absolute values of EC measurements in the P1-TZ-P2 region between the data presented here and those reported previously are likely a function of the hydrogeology of the system, where seasonal variations in primarily evaporation, as well groundwater influx and local injection influence water levels and EC values^12,22,57,58,64–66^. Importantly, the persistence of a consistent spatial gradient over more than a decade since the initial measurements collected by Otálora et al.^22^ implies the hydrogeological factors driving gradients in the chemistry of the system are likely resilient to perturbation.

To our knowledge, the measurements presented in this study from the main lagoons of Puquios 3 and 4 and their associated peripheral ponds are the first assessment of spatial heterogeneity and stratification in EC values within this part of the Salar de Llamara system. Limited point measurements of brines in Puquio 4 made by Rasuk et al.^30,41,43^ during summer months include an EC measurement (191 mS/cm) that was higher than our more recent observations in Puquio 4 (average of 150 mS/cm, n = 21; Fig. S1). Importantly, our new dataset documents a high degree of spatial heterogeneity even within a single lagoon, highlighting the importance of increased sample density in extreme environments that likely contain large gradients across relatively short distances. For example, the main lagoon and peripheral ponds of Puquio 4 (Fig. 2a, Supplemental Fig. S1) contain some of the highest EC measurements in the entire system, and the peripheral ponds exhibit an EC range that approximates the values observed in the 440 lagoons of the Transition Zone (Fig. 2c) over a shorter distance (Fig. 2).

Stratification of the brines was also variable across the Puquios system. On average, normal stratification was observed in the main lagoons of Puquios 1 and 2, the ponds in the Transition Zone—similar to that observed by Otálora et al.^22^—as well as the peripheral ponds in the vicinity of Puquios 2 and 3 (Fig. 2, Supplemental Fig. S1, Supplemental Table S1). However, normal stratification of the brine column was not a feature identified consistently across the system. For example, inverse stratification was observed in the main lagoons of Puquio 3 and 4, as well as the peripheral ponds near the main lagoon of Puquio 4 (Fig. 2, Supplemental Fig. S1, Table S1), suggesting that processes beyond density stratification are likely to be important drivers of water column dynamics. When assessing stratification on a point-by-point basis, rather than using values averaged across a given lagoon, additional areas of inverse stratification were also observed. In addition to stratification in TDS, Otálora et al.^22^ found varying degrees of normal stratification with respect to temperature, dissolved oxygen and pH values. While discussion of these parameters is beyond the scope of the present study, it is expected that spatial trends in these parameters would be likely observed in our dataset as well. Beyond the Puquios, gradients in environmental conditions have been linked to mineral precipitation in other modern and ancient extreme environments, such as the salt works at Guerrero Negro^67–69^, the EMISAL salt works in Egypt^70,71^, and the Messinian evaporite deposits^59^. Such extreme conditions typically subject resident biota to wide fluctuations in salinity, as well as temperature, pH, dissolved oxygen, total dissolved solids and redox potential^72,73^. Spatial variability in these biologically-relevant parameters have significant implications^12,14,20,22,30,41,43,64,65^ for the distribution of microbiological communities and mineral precipitation that is observed within the various chemical niches produced by the environmental gradients that characterize the Puquios ecosystem.

Gradients in electrical conductivity appear to drive spatial distributions of abundances and diversity of the free-living biota in the Puquios ecosystem (Fig. 7). Primary producers like phytoplankton and phytobenthos exhibit high abundance throughout the lagoons (Fig. 3). Abundance of phytobenthos showed a significant negative linear relationship with EC, decreasing in Puquios 2, 3 and 4 (Supplemental Fig. S2). No significant relation between EC and total phytoplankton abundance was observed (Supplemental Fig. S2) as total abundance of phytoplankton remains high throughout the EC gradient because of a shift to halotolerant taxa. For example, halotolerant diatoms such as *Staurosira* end *Nitzschia*, both on the bottom and in the water column, dominate in lagoons with the highest EC values (Puquios 2 and 4). Other abundant diatoms in these puquios include Fragilariales (i.e., *Staurosira and Staurosirella*), which are common in aquatic environments of the arid Andes of Chile and considered indicators of early successional stages^74^. On the other hand, cyanobacteria in the puquios occur in highest abundance in the shallowest lagoons with lower EC values (Puquio 1 and Puquio 3), where they conduct anoxygenic photosynthesis^75^ and share the ability to use elemental sulfur for anaerobic dark respiration with many archaeobacteria^76^, thus, favorably exploiting the local conditions in these puquios. Diversity indices for both phytoplankton and phytobenthos, show negative linear relationships with EC, as less halotolerant species are excluded with increasing EC.

Consumers (zooplankton and zoobenthos) were more variable than primary producers in their response to increasing electrical conductivity (Fig. 7). Zooplankton abundance has a positive linear relationship with EC, whereas zooplankton diversity index (H′) shows no relationship with EC (Supplemental Fig. S2). This pattern of increasing abundance and no change in diversity can be explained by the dominance in the water column of the branchiopod *Artemia franciscana* in all four main lagoons. Originally from North America, the presence of this species in the Puquios, and northern Chile in general, is strongly linked to bird migratory flyways^77^. The cyst of *Artemia* is highly resistant to extreme environmental conditions, while the motile stages are among the best osmoregulators known^78^, demonstrating their adaptability to the harsh environmental conditions that characterize extreme environments. In contrast, zoobenthos abundance showed no linear relationship with EC, with the higher abundances in Puquios 1 and 4 and lower abundance in 2 and 3 (Supplemental Fig. S2). Benthic dominance of the gastropod *Heleobia* in Puquio 1 was conspicuous. This gastropod has been described in saline lagoons in northern Chile and it is usually associated with aquatic macrophytes like *Chara* sp, which in the case of the Salar de Llamara, were only observed to be present in Puquio 1^79^. Larval stages of dipterans were dominant in Puquios 2–4, like Ephydridae, known for its broad tolerance to osmotic conditions^80^. Unlike zoobenthos abundance, zoobenthos diversity shows a negative linear relationship with EC (Supplemental Fig. S2).

The abundance and diversity of microbial communities in the brines and E1 layer of the gypsum structures in the Puquios also appear to be influenced by EC (Fig. 7). Although direct cell counts were not made, relative abundances of functional groups showed changes across the EC gradient. In particular, the variability in the abundance of functional groups in brine microbial communities was related to a shift from Alpha-based (Rhodobacterales and Sphingomonadaes) to Gamma-based (Chromatiales) anoxygenic phototrophy (Supplemental Fig. S3), a halotolerant group which grow autotrophically by utilizing energy derived from the oxidation of elemental sulfur and reduced inorganic sulfur compounds^81^ The E1 layer of low EC puquios was associated with higher relative abundances of nitrate and sulfate reducers and sulfur oxidizers, whereas the E1 layer of high EC puquios was associated with higher abundances of halophilic and halotolerant oxygenic and anoxygenic phototrophic genera (Figs. 4e, 7, Supplemental Fig. S4c,d). Changes in community structure of free-living bacteria and endoevaporitic bacteria along salinity gradients have been well documented in other similar environments and are likely related to the dominance of more halo-tolerant groups^41,82–86^.

Spatial gradients in EC also appear to be the dominant driver of microbial diversity. Diversity within the brines as measured by Shannon’s index (H′) shows a general decreasing trend with EC (Puquio 1: 3.9; Puquios 2 and 3: 3.1; and Puquio 4: 2.6) and differences in the brine bacterial communities were correlated to electrical conductivity in the principal coordinate canonical analysis (Fig. 4c). Microbial diversity in the E1 layer, as measured by the Shannon index, was essentially unchanged across all four puquios (Puquio 1: 4.6; Puquios 2: 4.7; Puquio 3: 4.7; and Puquio 4: 3.5). However, the structure of the community did change with increasing EC according to the principal coordinate canonical analysis (Fig. 4d), supported by a shift in the relative abundances of chemotrophs to phototrophs in higher EC puquios, as described above. The impact of EC on the E1 layer communities suggests that pore water in the subaerial structures is fed by the lagoons via capillary action^87,88^. These porewaters are likely further impacted by evaporation as they travel up the structures, raising the EC beyond the initial composition of the waters sourcing them.

High resolution bottom type mapping in the Puquios provided new insight into the spatial heterogeneity of mat morphology and associated mineral assemblages, and their relationship to EC values in this heterogeneous system (Fig. 7). In the case of the Puquios, bottom types were previously classified as either microbial or crystalline (*sensu*^22^). However, our high-resolution assessment indicates that within these major classifications, a variety of both microbial and crystalline bottom types contain a diverse suite of minerals and morphologies that occurs within the gradient of EC values. This observation suggests the system is more heterogeneous than previously appreciated. Similar analyses conducted in other extreme environments have demonstrated the utility of such high-resolution observations, where spatial context of the diverse bottom types with environmental parameters provides insight into factors that impact and often segment these systems^8,68^. Within the Puquios, the gradient in EC values appears to be the overarching mechanism that subdivides the bottom type morphologies and minerals precipitated. Where EC values are relatively low, as in Puquio 1 (Figs. 2, 5g), flocculent microbial mats are abundant (Fig. 5a,g). Mineral precipitation within these includes carbonates, clays, and fine-grained, granular gypsum (Fig. 6) that are characterized by irregular and lenticular shape that have been interpreted to be typical of microbial precipitates^68,69,89^. In many instances, these low to moderate EC environments contain carbonate and authigenic clay forming within EPS matrices and along cell sheaths, that is consistent with microbial influence^90,91^. With the increasing EC values, mats become more coherent and laminated across both the Transition Zone (Fig. 5a,b,g, Supplemental Fig. S6a–c) and within the main lagoon of Puquio 3 (Fig. 5c,g), where microbial influences are the main driver of mineral precipitation (Table 1).

**Table 1:** Summary table showing main bottom type, dominant mineralogy, and interpretation of microbial influence in precipitation across the Puquios.

	Main bottom type	Mineralogy	Microbial influence
Puquio 1	Floccules with gypsum in mats around the margins	Gypsum (fine-grained/granular)	Microbial
Transition Zone	Various microbial mats	Gypsum (granular to laminated)	Microbial
Puquio 2	Spar	Gypsum (selenite crystals)	Physicochemical with microbial influence around the margins
Puquio 3	Pinnacles	Gypsum	Microbial and physicochemical
Puquio 4	Spar	Gypsum (selenite crystals)	Physicochemical

In contrast, when brines have relatively high EC values, such as those observed in Puquio 2 and in Transition Zone ponds adjacent to the southwestern margin of Puquio 2 (Figs. 2, 5g, Supplemental Fig. S1) the pond bottoms are dominated by gypsum spar whereas microbial mats are rare (Fig. 5a). However, thin, dark biofilms, and/or oxide coatings on crystal surfaces are common (Fig. 6i,m). Similar to Puquio 2, Puquio 4 is also characterized by relatively high EC values (Figs. 2, 5g, Supplemental Fig. S1) and gypsum spar and botryoids are the dominant bottom types (Fig. 5e,f, Supplemental Fig. S6f). The prismatic euhedral crystals in Puquios 2 and 4 are typical of gypsum precipitates formed in in other high EC environments^70,71^ and are interpreted to form through physicochemical mechanisms (Table 1). This observation of an EC-related control is consistent with variability in mineralogical characteristics observed other evaporitic environments (e.g.^59,68,70,71,92^). As a result, these findings suggest that variability in the morphological expression of bottom types is a useful visual representation of both the environmental gradients and likely spatial heterogeneity in mineral deposition within these ecosystems.

The dynamic interplay between chemical, biological and geological aspects of the system may foster adaptability in community structure and function. For example, the Invierno Altiplánico that occurred in the Atacama Desert in January–February 2017 is hypothesized to have impacted both water levels and microbial communities in Puquio 1. Higher water levels after the Invierno Altiplánico likely filled the pore spaces of the subaerially exposed structures, reduced available oxygen in the environment that fosters endoevaporitic microbial communities, and produced the observed enrichment in the anaerobic microbial population in Puquio 1 samples. The surface water level therefore is an important determinant of the shift from a subaerial to a subaqueous lifestyle as was previously observed^41^. Considering that EC has an indirect correlation with surface water level in the Puquios (R^2^ = 0.50–0.83), seasonally variable water levels in Puquio 1 may drive the relationship between EC and microbial community structure in Puquio 1, distinguishing it from similar endoevaporitic communities near Puquios 2, 3 and 4. Similar seasonal differences in anaerobic Deltaproteobacteria abundances were observed between summer and winter samples in a previous study^41^ and between the depths analyzed in subaqueous niches in March 2012^14^.

Given the reported diversity of water chemistry, biota, minerals, and structures, of other microbial Andean ecosystems within Chile, Bolivia, and Argentina^26,93^, we hypothesize that heterogeneity similar to that observed in the Puquios is likely to be a common characteristic of extreme environments and that seasonal environmental variability, changes in electrical conductivity driven by seasonal cycles of evaporation, and groundwater flow are likely to impact all of the ecological niches to varying degrees at different times of the year. This concept is critical when characterizing such ecosystems, since a single sample is likely not representative of the entire system. As a result, more attention should be given to data collection through space and time when interpreting the significance of biogeochemical processes in these extreme environments.

In summary, the diagram shown in Fig. 7 provides a conceptual framework highlighting trends in the biological and geological components of the Puquio system across an increasing gradient in electrical conductivity. This figure shows that despite a shift to more halotolerant species with increasing electrical conductivity, key metabolisms such as phototrophy are maintained. We also observe a succession from less energy yielding to more energy yielding metabolisms (e.g. heterotrophy to autotrophy, anaerobic to aerobic), in order to handle increasing osmotic stress^94^. These observations suggest that metabolic redundancy and diversity inherent in these biological communities may contribute to the resilience of the system to harsh poly-extreme conditions. Moreover, seasonal changes in precipitation, evaporation, temperature, and climatic conditions drive temporal variability in environmental conditions^22,57,58^ that are at least as extreme as the spatial heterogeneity documented in the Puquios. Such spatio-temporal heterogeneity presents significant challenges to survival in the Puquios. Thus, we propose that heterogeneity in all aspects of the Puquios system may lead to ecological flexibility, including functional redundancy and adaptability^95^, both of which may be fundamental for the ecosystem to thrive in some of the harshest conditions on Earth.

## Conclusions

Our study documents a previously unrecognized degree of spatial heterogeneity in water chemistry, free living biota, microbial communities and bottom types in the Puquios system. Biological and geological variability appear to be predominantly driven by heterogeneity in electrical conductivity. Results demonstrate the importance of high-resolution measurements, collected across multiple disciplines, from extreme environments that are inherently heterogeneous. Moreover, it is likely that observations from a single season do not capture the full range of variability, and enhanced spatio-temporal heterogeneity is expected as seasonal changes in environmental conditions fluctuate. Inherent multidisciplinary heterogeneity may be key to the success of these microbial ecosystems, providing flexibility that allows them to reorganize after disturbances and persist in extreme conditions at the edge of habitability.

## Methods

### Quantification of benthic and pelagic biota

Biotic survey of water column and benthic organisms was performed at 5 sites in austral summer 2017 within each of the 4 Puquios. At each site 3 replicate samples were obtained. For zooplankton and phytoplankton 36 L of water was passed through a 35 µm and 60 µm sieve respectively by means of an electrical immersion pump powered by a portable 12 V battery. Each sample was summarized and introduced into a 50 mL Falcon tube and fixed using 2% Lugol for phytoplankton and 5% diluted formalin for zooplankton. For phytobenthos, an 875 mm^2^ cylindrical plexiglass tube was gently introduced into the top bottom 1 cm layer of the substrate and all material collected stored into a 15 mL Falcon tube and fixed using 2% Lugol. Zoobenthos samples were collected using a 1256 mm^2^ cylindrical plexiglass tube that was also introduced into the top 1–2 cm layer of substrate and all collected material stored into an 80 mL plastic container and fixed with 5% diluted formalin. Phytoplankton and phytobenthos quantification analyses considered APHA^95^ methods 10200 C, D, E, F and 10300 C, and for identification several sources were consulted (e.g.^96–105^). Zooplankton and zoobenthos quantification analyses considered APHA^95^ methods 10200 G and 10500 A, C and Woelf et al.^106^ and for identification several specialized sources were consulted (e.g.^107–112^). A linear regression analysis of the relationship between abundance as well as diversity (Shannon index H′) and electrical conductivity was completed using Prism version 8.4.2. from GraphPad Software (San Diego, Ca, USA).

### Characterization of microbial communities

Bacterial communities were collected from subaerial gypsum structures at 3 sites from each of the four Puquios (n = 12) using a 20 cm sediment core device in March 2017. A sample of the microbial mat inhabiting the gypsum structure was collected from the E1 ‘green layer’. A 1 L brine sample was collected from the water surrounding each subaerial gypsum structure for comparison between the planktonic microbial community and the microbial mat community. The E1 from each core sample of the subaerial gypsum structures was isolated in the lab for DNA extraction and the brines were filtered by a nitrocellulose membrane with a 0.22 µm pore size (Merck-Millipore, Germany) to collect the cells as it was reported before^113^. The filter was then folded and placed in a tube with a hypertonic lysis buffer solution (25.7% sucrose, 50 mM TRIS–HCl, 40 mM EDTA) and was kept at − 20 °C up to later processing. DNA was obtained using the AllPrep Qiagen RNA/DNA Isolation Kit (Qiagen, USA). The extraction yield and DNA purity were determined by means of gel electrophoresis and UV–Visible spectrophotometry (Nanodrop, Thermo, Germany).

The V1-V3 variable region of the 16S rRNA gene were amplified in a PCR thermocycler using the primers 27f. (5′-AGAGTTTGATCCTGGCTCAG-3′)^114^ and 534r (5′-ATTACCGCGGCTGCTGG-3′)^17^ for bacteria with a barcode on the forward primer. PCR was performed using the HotStarTaq Plus Master Mix Kit (Qiagen, USA) under the following conditions: 94 °C for 3 min, followed by 28 cycles of 94 °C for 30 s, 53 °C for 40 s and 72 °C for 1 min, after which we performed a final elongation step at 72 °C for 5 min. PCR products were visualized in 2% agarose gel to determine the success of amplification and the relative intensity of the bands. Multiple equivalent samples of PCR products were pooled together in equal proportions based on their molecular weight and DNA concentrations. Pooled samples were purified using calibrated Ampure XP beadsto prepare a DNA library by following the Illumina TruSeq DNA library preparation protocol. Sequencing was performed at MR DNA (www.mrdnalab.com, Shallowater, TX, USA) on a MiSeq, following the manufacturer’s guidelines. The sequence data were processed using a proprietary analysis pipeline (MR DNA) with the following steps: the barcodes were eliminated from sequences, then sequences < 150 bp and those with ambiguous base calls were removed. Then, sequences were denoised, OTUs generated and chimeras removed. The reads were added to the Sequence Read Archive (SRA) National Center for Biotechnology Information (NCBI), (http://trace.ncbi.nlm.nih.gov/Traces/sra/). Project numbers—BioProject accession: PRJNA659028 (Puquios of the Salar de Llamara Raw sequence read); BioSample: SAMN15924840 (A multidisciplinary evaluation of spatial heterogeneity in the Puquios of the Salar de Llamara, Atacama Desert, Northern Chile); SRA: SRR12536357 (Puquios of the Salar de Llamara Atacama Desert).

The sequences were processed further by means of the QIIME (Quantitative Insights Into Microbial Ecology) pipeline^115^: Raw sequences were filtered by base quality score, average base content per read and GC distribution in the reads. Reads that did not cluster with other sequences, i.e. singletons (abundance < 2) were removed. Chimeras were also removed using the UCHIME program^116^. The pre-processed sequences were finally grouped into operational taxonomic units (OTUs) using the clustering program UCLUST at a similarity threshold of 0.97^116^. The pre-processed reads were used to identify the OTUs using QIIME and aligned the representative sequences against the Greengenes core set reference database using PyNAST^115^. A representative sequence for each OTU was classified using RDP classifier and the Greengenes OTU database. The alpha-rarefaction was then calculated by means of the “core_diversity_analysis” command and standardized the number of sequences to the smaller sample size by means of Chao 1 (2599 sequences). The rarefacted data using the Primer-7 (Primer-E) software^117^ was used to determine the beta diversity and plot the main coordinates graphs. Non-metric multidimensional scaling (NMDS), Principal Coordinate (PCO) and Principal Coordinate Canonical analysis (CAP) were used to build a constrained ordination of the OTUs or groups of other taxonomic levels, based on the environmental data collected at the time of sampling^113^. Additionally, PICRUSt^118^ was used to predict functional composition of the metagenomes from 16S data.

### Brine chemistry used in nMDS plots

Brine samples adjacent to the subaerial gypsum structures were collected so correlation to the bacterial community profiles of E1 layers could be made. Cation concentrations were measured using an atomic absorption spectrophotometer (AAnalyst 800; Perkin-Elmer, Norwalk, CT, USA). Sulfate and chloride contents were measured by gravimetric and Mohr method^119^, respectively.

### In situ electrical conductivity measurements

In situ measurements of electrical conductivity (EC) were collected over the course of three days in austral summer 2017 using a HI 9829 multimeter (Hanna Instruments, Rhode Island, USA). The EC probe was calibrated to known standards of EC values (HI7031L and HI7035, Hanna Instruments, Rhode Island USA) each morning before the start of field sampling. Sample locations were collected with a Garmin GPSMap 78 s. For EC, the manufacturer specifications for accuracy is ± 1 µS/cm or ± 1% of the reading, whichever is larger. Measurement resolution is 1 µS/cm, and the range is 0–200 mS/cm. At each location, both a surface measurement and bottom brine measurement were collected. Surface measurements were collected first at about 5 cm depth in order to minimize disruption of any brine stratification, with the probe oriented parallel to the water surface. Bottom brines were measured second by slowly lowering the probe to the bottom of the lagoon and laying it horizontally. Bottom measurement collection was delayed until EC values stabilized, often 15–20 s. These field measurements, brine depths, and GPS locations were merged into a spreadsheet and their spatial distributions mapped in ArcGIS Pro. The data were evaluated by calculating the average and standard deviations, as well as calculating the interquartile range (IQR) using the exclusive quartile calculation in Microsoft Excel (Office 365). Stratification was calculated following the methods of Babel^59^, where surface measurements are subtracted from bottom measurements. Normal stratification is then defined as bottom measurements being greater than surface measurements, and inverse stratifications are defined as bottom measurements being lower than surface measurements.

### Bottom type mapping and microbe-mineral interactions

To distinguish physio-chemical substrates in the Puquios from substrates that are products of benthic organisms, we adopted a multiscale approach. At the macroscale, broad facies patterns were mapped using aerial imagery; these bottom type maps were refined using comprehensive ground truthing in austral summer 2017. This mapping approach identified principal bottom types based on upper few centimeters of the substrate surface across the Puquios system (Supplementary Figs. S5, S6). At the mesoscale, hand samples of the different bottom types were described (Supplemental Table S4). At the microscale, crystal morphologies in context with surrounding matrices were analyzed in the lab using scanning electron microscopy (SEM) with electron dispersive spectroscopy (EDS). This comprehensive approach defined mapping units used to document spatial distribution of bottom types and distinguished between abiotic mineral structures and those mineral deposits influenced and/or induced by microbial mats.

High resolution drone images of the Puquios were georectified in ENVI 5.6 and loaded onto an iPad mini into Global Mapper Mapper Mobile, a powerful GIS data viewing and field data collection application for iOS. The software application uses the iPad’s GPS capability to provide location information for remote mapping projects. The iPad mini was used in the field during the January 2018 field campaign where drone imagery and shapefiles were loaded into the application and subsequently, multiple locations throughout and around each Puquio, including all accessory ponds were investigated. At each location, a photograph was taken, and the bottom type was identified and described.

SEM imaging was performed on a ThermoFisher Scientific Apreo Field Emission Scanning Electron Microscope fitted with a compound electrostatic and immersion final lens, low vacuum capability and Edax Octane Energy Dispersive X-Ray Spectrometer (EDS) with 60 mm Silicon Drift Detector at Smithsonian National Museum of Natural History.

## Supplementary Information

Supplementary Legends.

Supplementary Figure S1.

Supplementary Figure S2.

Supplementary Figure S3.

Supplementary Figure S4.

Supplementary Figure S5.

Supplementary Figure S6.

Supplementary Figure S7.

Supplementary Table S1.

Supplementary Table S2.

Supplementary Table S3.

Supplementary Table S4.
