# Supplementary material for: Electrical conductivity as a driver of biological and geological spatial heterogeneity in the Puquios, Salar de Llamara, Atacama Desert, Chile
Source: Sci Rep. 2021 Jun 17;11:12769. doi: 10.1038/s41598-021-92105-2 (PMC8211675; doi:10.1038/s41598-021-92105-2)
Supplement: Supplementary file 1 — Supplementary Legends. [file 41598_2021_92105_MOESM1_ESM.docx]

**Supplementary Material for:**

**Electrical conductivity as a driver of biological and geological spatial heterogeneity in the Puquios, Salar de Llamara, Atacama Desert, Chile**

R.P. Reid^1,2^, A.M. Oehlert^1,2^, E.P. Suosaari^1,2,3^, C. Demergasso^4,5^, G. Chong^5,6^,

L.V. Escudero^6^, A.M. Piggot^2,7^, I. Lascu^3^, A.T. Palma^8^

**Supplemental Figure S1.** Box and whisker plot of electrical conductivity measurements separated by lagoon type. Lagoons labeled Puquio 1, 2, 3, and 4 refer to measurements collected within the limits of the largest and contiguous main lagoon in the system (see Fig. 1 in main text for map). Datasets labeled ‘peripheral’ refer to the smaller ponds located near the main lagoons of the system but are not directly connected to the main lagoon water body at the air-water surface. These lagoons can often have very different electrical conductivity than the main lagoons.

**Supplemental Figure S2.** Linear regression analyses of abundances and Shannon’s diversity indices (H’) against EC for the main biotic assemblages (phytoplankton, phytobenthos, zooplankton and zoobenthos) present in the bottom and water column of each puquio. In each case, the F value and the probability of the regression (P) are provided; P values less than 0.05 are considered significant. Figure was created using Prism version 8.4.2 from GraphPad Software. <https://www.graphpad.com>

**Supplemental Figure S3**. Histograms showing the relative abundance of 16S rRNA gene sequences in brine from each puquio assigned at the class and order level. Figure was created using Tableau Software <https://www.tableau.com/>

**Supplemental Figure S4.** Relative abundance of the dominant functional groups in the E1 layer from gypsum structures surrounding the puquios determined by 16S rRNA gene sequences. (a) Relative abundance of sulfate reducers from the class delta-proteobacteria (b) relative abundance of sulfur oxidizers from the class gamma-proteobacteria (c) relative abundance of anaerobic phototrophs from the phylum Chloroflexi and (d) relative abundance of aerobic phototrophs from the phylum Cyanobacteria. Figure was created using Tableau Software <https://www.tableau.com/>

**Supplemental Figure S5.** Principle bottom types in alphabetical order: a) *Black domes/black bulbs:* consolidated and gelatinous up to 5 cm in relief; b) *Black mantle:* hard encrusting surfaces occurring as meter-scale domal, flat, or platy features; c) *Black pinnacle mat:* small (< 5 mm) pinnacles growing vertically; d) *Black semi-cohesive mat:* soft, loosely amalgamated mat that easily disaggregates; e) *Blister:* semi-dry, vermiculated surface morphology; f) *Botryoids:* rounded globules of mineral precipitation; g) *Brown gel film mat:* thin gel consistency, often a superficial coating on botryoid surfaces; h) *Brown smooth mat:* < 1 cm thick semi-cohesive mat with a smooth surface, pale-yellowish-gray to brown; i) *Fine-grained precipitate:* dried, fine, powdery white precipitate; j) *Flaky sheets/cracked crust:* dried, thin precipitate laminae, often with sub-surface air pockets; k) *Floccules:* non-cohesive organic material; l) *Grass:* herbaceous plants with narrow leaves sometimes coated in precipitation (m); n) *Network on bulbous mat*: thick, gelatinous bulbs of microbial mat up to 4 cm in diameter, orange to brown in color, with a superficial thin web-like network coating; o) *Orange carpet*: soft, shaggy carpet tufts of mat, orange to pink in color with darkened grey to black patches; p) *Orange gel mat*: < 1 cm thick cohesive mat with a smooth surface; q, r) *Orange gel mat with black pinnacles*: < 1 cm thick smooth and cohesive mat with small < 5 mm black pinnacles growing vertically; s) *Orange/brown pustules*: rough mammilate surface with rubbery-like pustules < 3 cm in diameter; t) *Orange/brown bulbous mat*: thick, gelatinous bulbs of microbial mat < 4 cm in diameter; u) *Pink smooth mat*: <1 cm thick smooth cohesive mat; v, w) *Pinnacles:* well-cemented, < 1 cm in relief pinnacles, peach to orange in color when submersed or white when subaerial; x) *Platy*: aggregations of botryoids formed as brecciated platy layers, sometimes coated with thin brown film; y, z, aa, ab) *Spar crystals:* crystals with readily discernible faces, light in color, typically 1-3 cm in length.

**Supplemental Figure S6.** Maps of Principle Bottom Types (PBT) showing Puquio 1 and 2 a) bottom types, and b) side types; Puquio 3 c) bottom types, and d) side types, and Puquio 4 e) bottom types and f) side types. Descriptions can be found in the figure caption of Supplemental Figure 1. Each colored dot represents a field observation, colored coded as in legend. Dots not associated with the major lagoons represent observations in smaller peripheral ponds surrounding the main Puquios. Maps were generated in Global Mapper v20.1 <https://www.bluemarblegeo.com/>

**Supplemental Figure S7.** Examples of principle bottom types collected throughout the Puquios. (a) Puquio 1 - floccules, (b) transition zone - black domes, (c) transition zone – orange/brown bulbous mat, (d) Puquio 2 - spar, (e) Puquio 3 - pinnacles, (f) Puquio 4 - spar.

**Supplemental Table S1.** Data collected using the Hanna meter from the Puquios in November 2017.

**Supplemental Table S2.** Number of taxa present and Shannon’s diversity index (H’) at each study point of each Puquio for all the benthic and water column assemblages.

**Supplemental Table S3.** Data table of chemical parameters measured during bacterial community sampling of gypsum subaerial structures and brines.

**Supplemental Table S4.** Description of microbial samples collected from the Puquios. Seven of the thirteen samples were collected from Puquio 1 as a result of the heterogeneity between smaller ponds. Three samples were collected from Puquio 2. Two samples were collected from Puquio 3 and one samples was collected from Puquio 4. The samples highlight the variability of microbial mats and bottom types throughout the Puquios. These samples were selected before the bottom types in the previous section were defined, but serve as examples of seven of the designated bottom types, as indicated.
