# Supplementary figures and images for: Electrical conductivity as a driver of biological and geological spatial heterogeneity in the Puquios, Salar de Llamara, Atacama Desert, Chile
Source: Sci Rep. 2021 Jun 17;11:12769. doi: 10.1038/s41598-021-92105-2 (PMC8211675; doi:10.1038/s41598-021-92105-2)

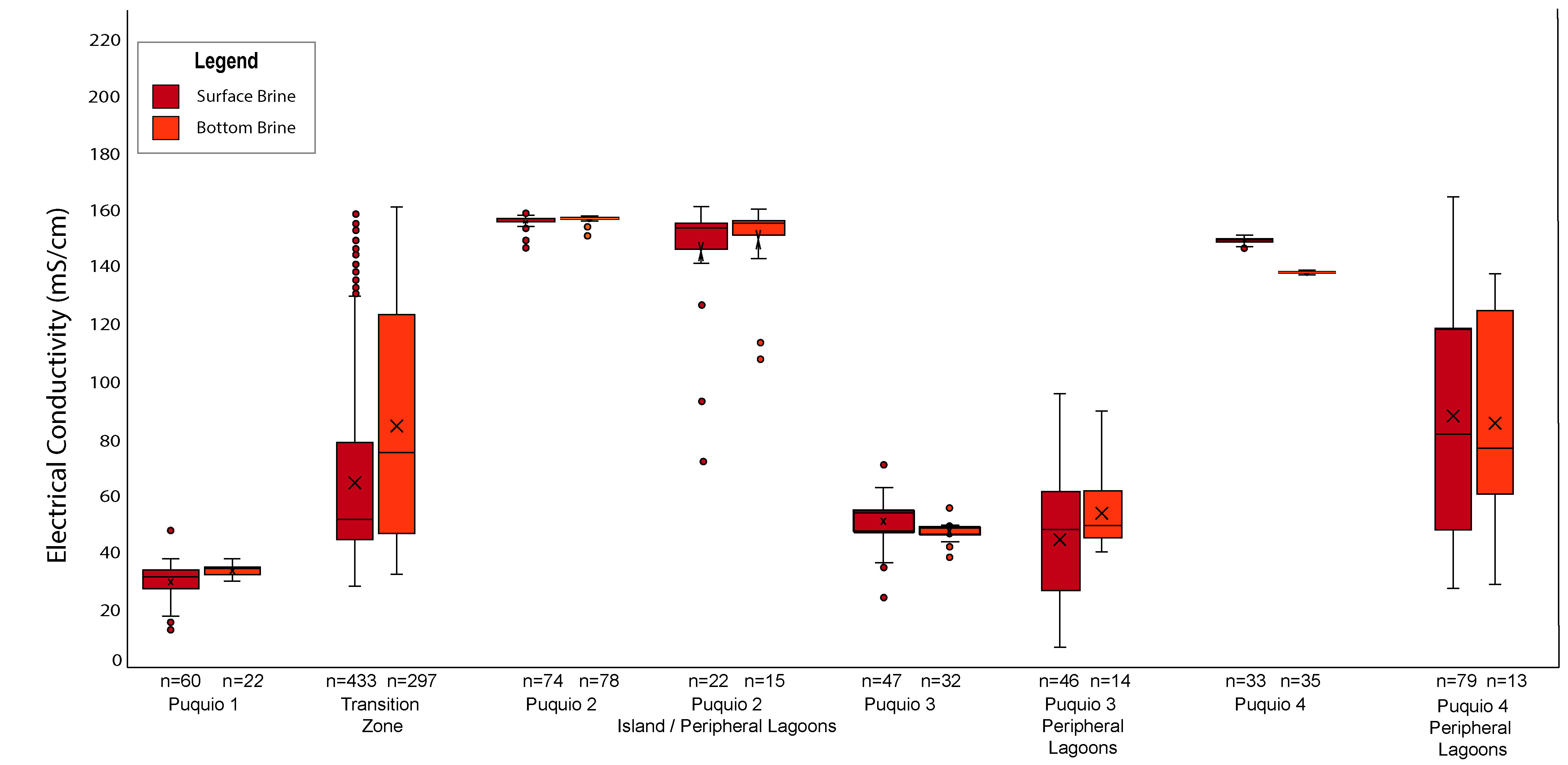

Supplement: Supplementary file 2 — Supplementary Figure S1. [file 41598_2021_92105_MOESM2_ESM.jpg]

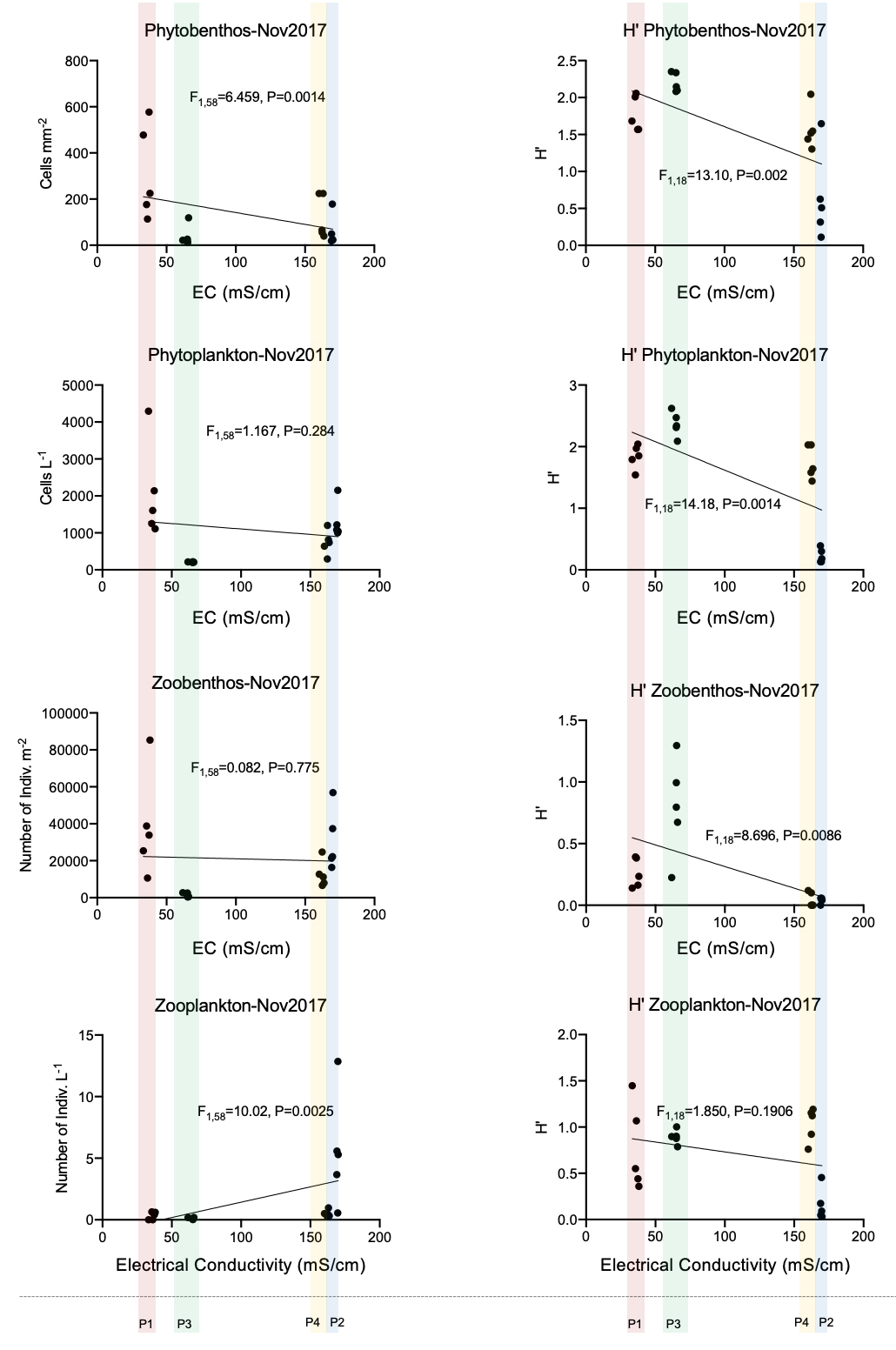

Supplement: Supplementary file 3 — Supplementary Figure S2. [file 41598_2021_92105_MOESM3_ESM.jpg]

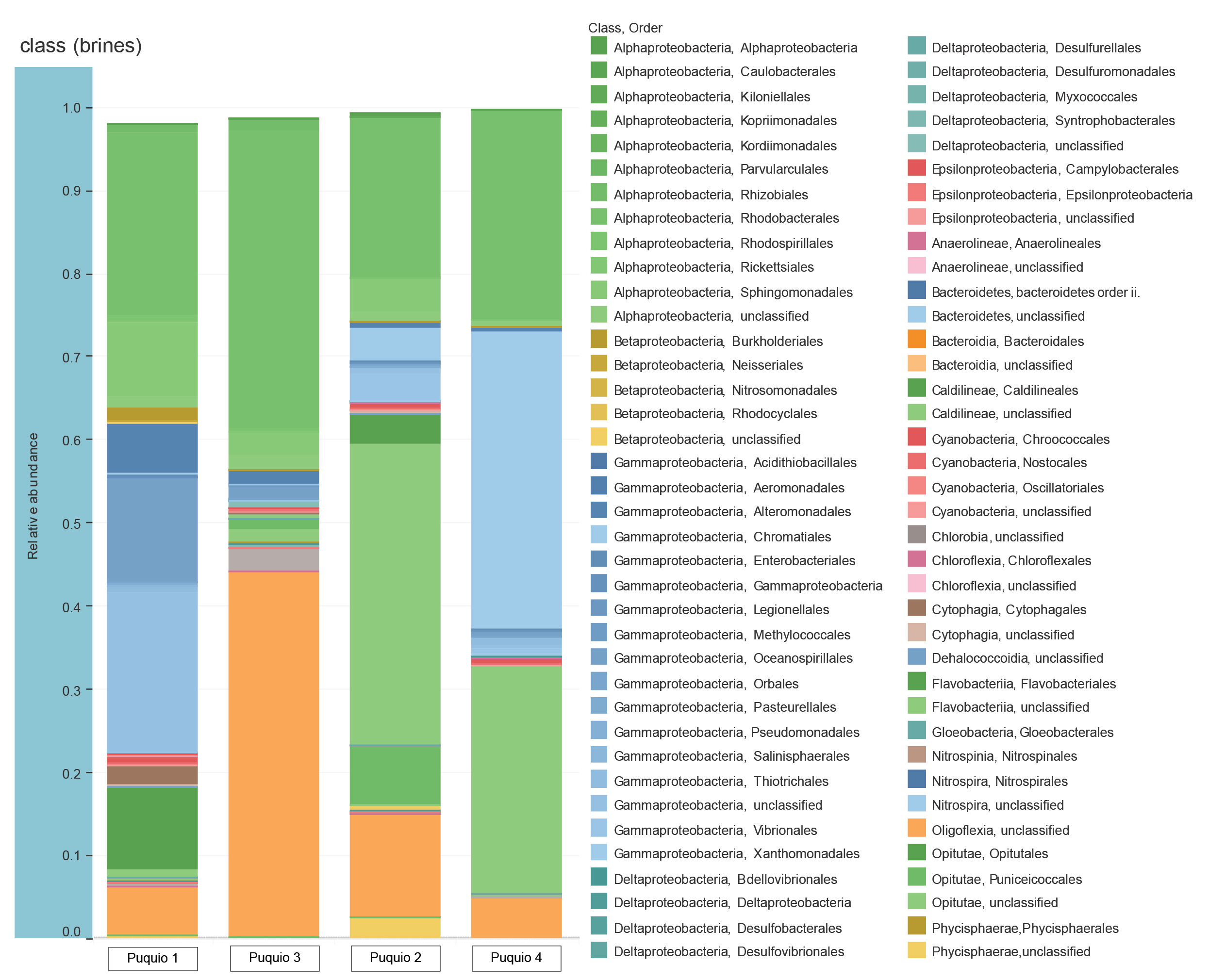

Supplement: Supplementary file 4 — Supplementary Figure S3. [file 41598_2021_92105_MOESM4_ESM.jpg]

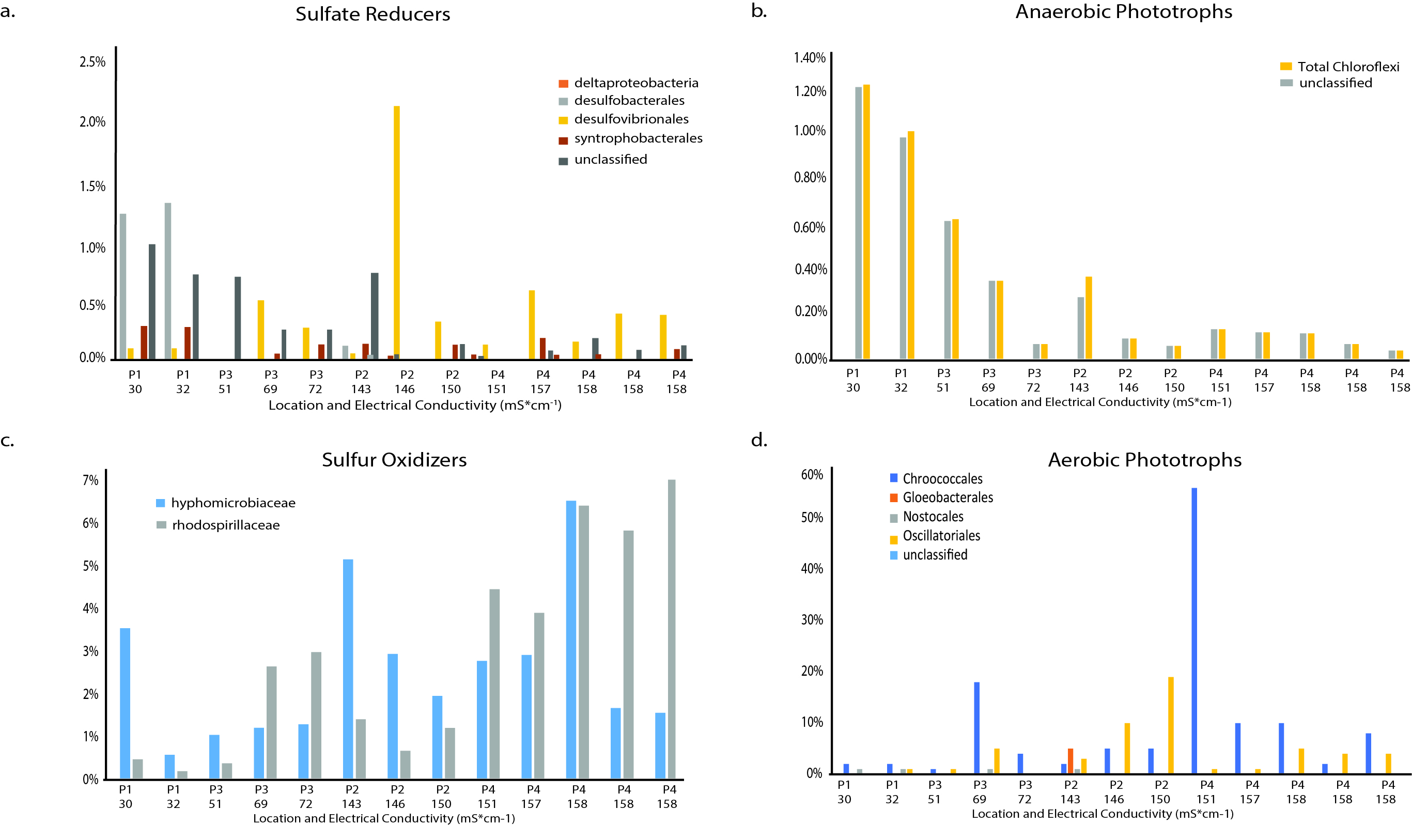

Supplement: Supplementary file 5 — Supplementary Figure S4. [file 41598_2021_92105_MOESM5_ESM.jpg]

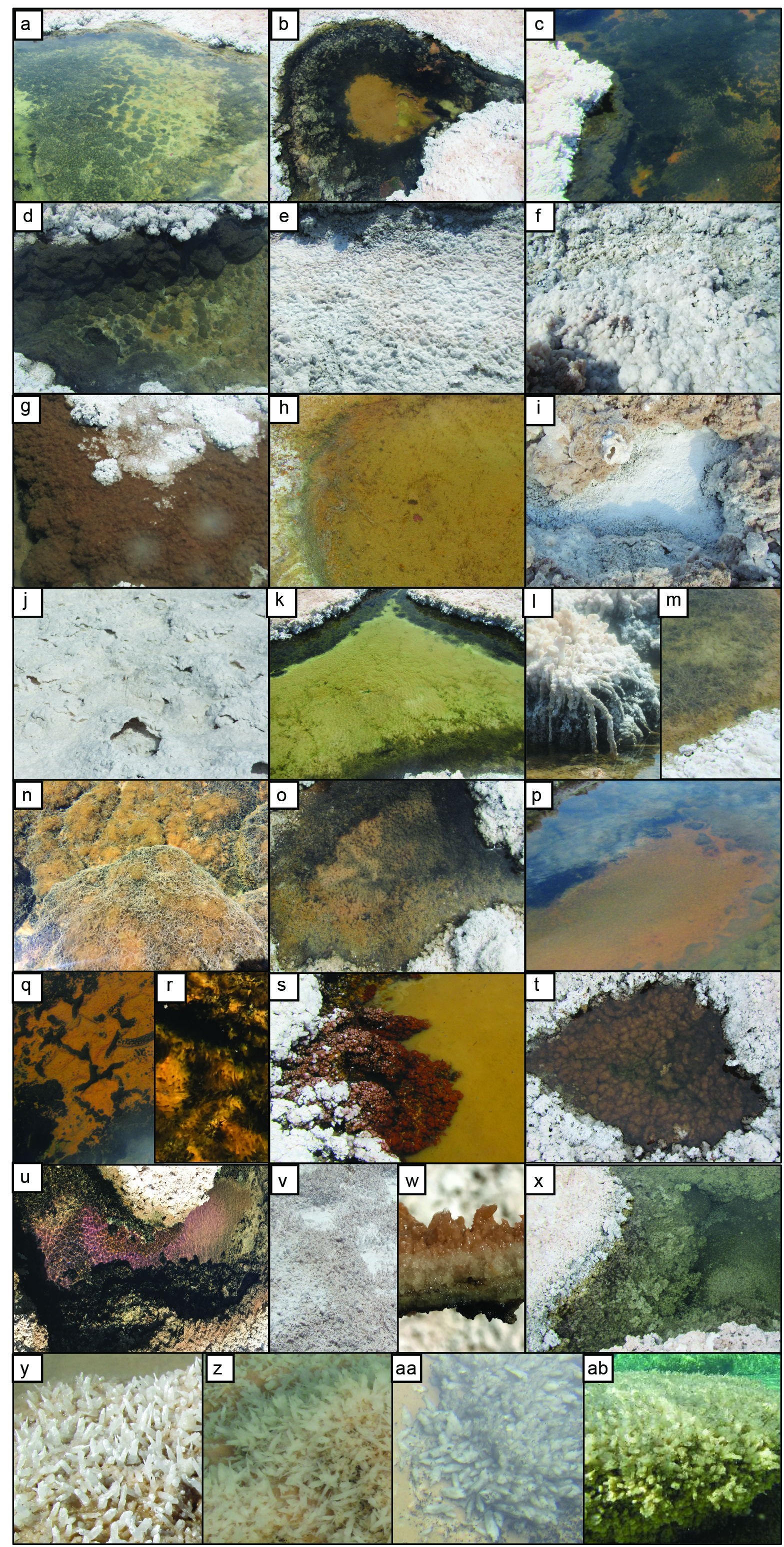

Supplement: Supplementary file 6 — Supplementary Figure S5. [file 41598_2021_92105_MOESM6_ESM.jpg]

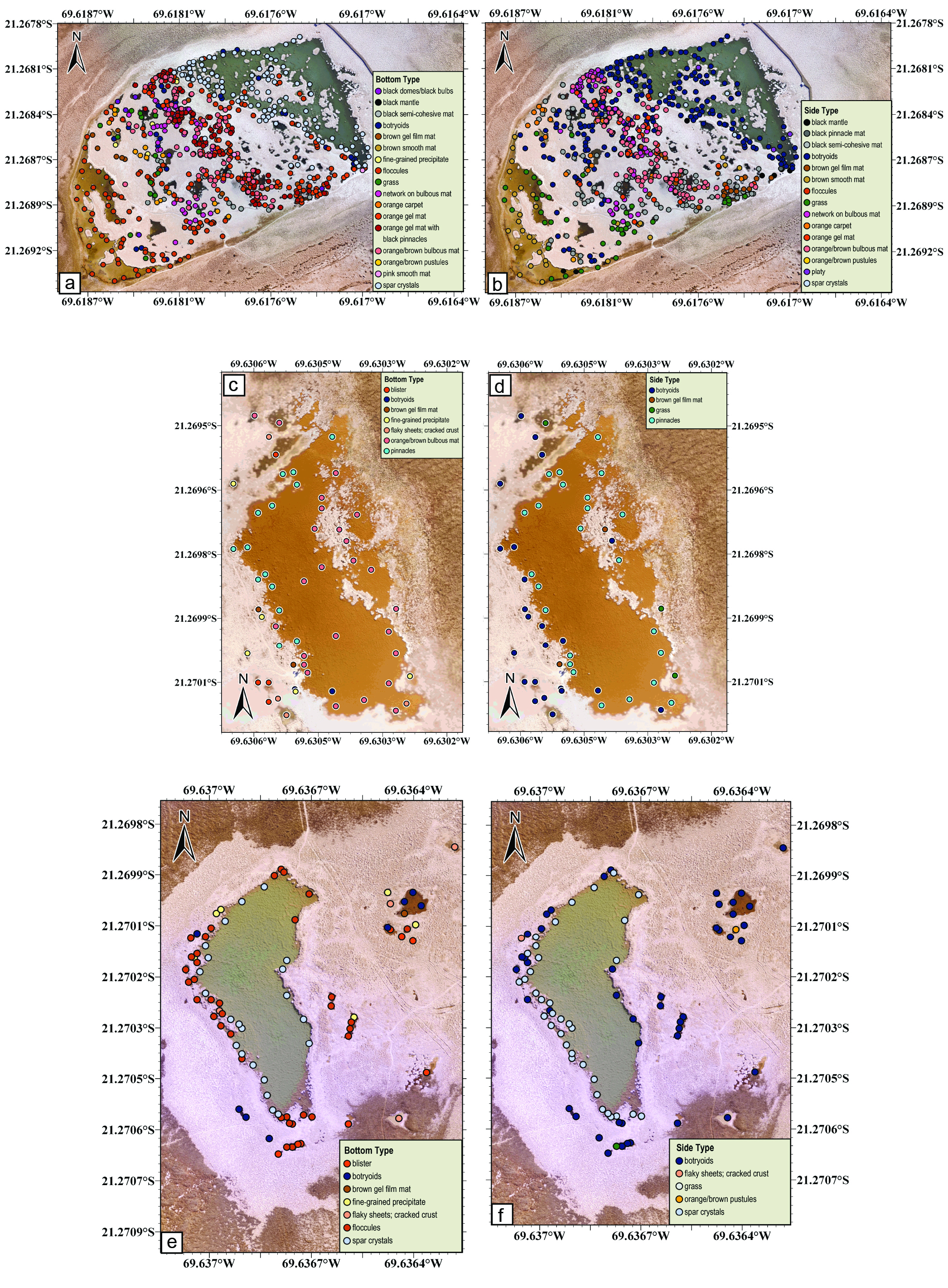

Supplement: Supplementary file 7 — Supplementary Figure S6. [file 41598_2021_92105_MOESM7_ESM.jpg]

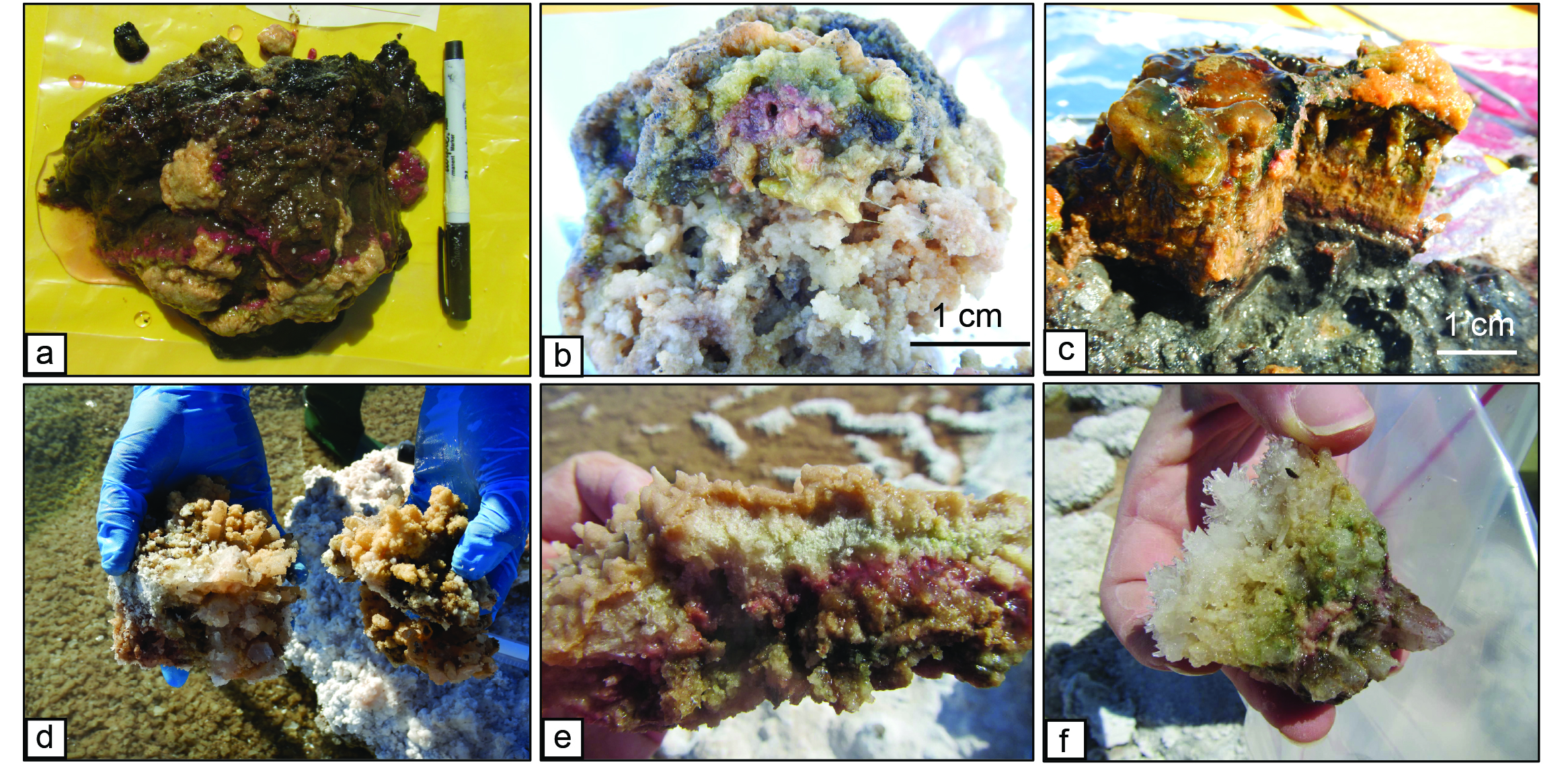

Supplement: Supplementary file 8 — Supplementary Figure S7. [file 41598_2021_92105_MOESM8_ESM.jpg]
